# Commensal Lactobacilli Metabolically Contribute to Cervical Epithelial Homeostasis in a Species-Specific Manner

**DOI:** 10.1128/msphere.00452-22

**Published:** 2023-01-11

**Authors:** Nicole R. Jimenez, Jason D. Maarsingh, Paweł Łaniewski, Melissa M. Herbst-Kralovetz

**Affiliations:** a Department of Obstetrics and Gynecology, College of Medicine—Phoenix, University of Arizona, Phoenix, Arizona, USA; b Department of Basic Medical Sciences, College of Medicine—Phoenix, University of Arizona, Phoenix, Arizona, USA; University of Michigan Ann Arbor

**Keywords:** *Lactobacillus iners*, *Lactobacillus mulieris*, *Lactobacillus paragasseri*, *N*-acetylated amino acids, aromatic lactic acids, cervicovaginal health, global metabolomics, glycerophospholipids, organotypic 3D culture, vaginal microbiome

## Abstract

In reproductive-age women, the vaginal microbiome is typically dominated by one or a few *Lactobacillus* species, including Lactobacillus crispatus, Lactobacillus iners, Lactobacillus paragasseri, Lactobacillus mulieris, and Lactobaccillus crispatus, has been associated with optimal cervicovaginal health; however, much is still unknown about how other lactobacilli metabolically contribute to cervicovaginal health. We hypothesized that metabolites of each *Lactobacillus* species differ and uniquely contribute to health and homeostasis. To address this hypothesis, we utilized a human three-dimensional (3D) cervical epithelial cell model in conjunction with genomics analyses and untargeted metabolomics to determine the metabolic contributions of less-studied vaginal lactobacilli—*L. iners*, *L. paragasseri*, and *L. mulieris*. Our study validated that vaginal lactobacilli exhibit a close phylogenetic relationship. Genomic findings from publicly available strains and those used in our study indicated that *L. iners* is metabolically distinct from other species of lactobacilli, likely due to a reduced genome size. Lactobacilli and mock controls were distinguishable based on global metabolic profiles. We identified 95 significantly altered metabolites (*P < *0.05) between individual lactobacilli and mock controls. Metabolites related to amino acid metabolism were shared among the lactobacilli. *N*-Acetylated amino acids with potential antimicrobial properties were significantly elevated in a species-specific manner. *L. paragasseri* and *L. iners* shared aromatic, but not carbohydrate-derived, lactic acid metabolites with potential antimicrobial properties that may contribute to homeostasis of the cervicovaginal environment. Additionally, *L. iners* uniquely altered lipid metabolism, which may be a sign of adaptation to the cervicovaginal niche. Overall, these findings further elucidate the metabolic contributions of three key vaginal *Lactobacillus* species in gynecological health.

**IMPORTANCE** *Lactobacillus* species contribute to cervicovaginal health by their production of lactic acid and other antimicrobial compounds. Yet, much is still unknown regarding the metabolic potential of lesser-studied but common vaginal lactobacilli. Here, we used untargeted metabolomics coupled with our 3D cervical epithelial cell model to identify metabolic differences among vaginal *Lactobacillus* species (Lactobacillus iners*, Lactobacillus paragasseri*, and *Lactobacillus mulieris*) and how those differences related to maintaining homeostasis of the cervical epithelium. Human 3D cell models are essential tools for studying host-bacteria interactions and reducing confounding factors inherent in clinical studies. Therefore, these unique models allowed us to decipher the putative lactobacilli mechanisms that contribute to their roles in health or disease. Metabolic analyses revealed distinct profiles of each *Lactobacillus* species but also shared metabolic contributions associated with antimicrobial activity: amino acid metabolism, *N*-acetylated amino acids, and aromatic lactic acids. These patterns provided validation of metabolites associated with health in clinical studies and provided novel targets, including immunomodulatory and antimicrobial metabolites, for postbiotic therapies.

## INTRODUCTION

Studies of the vaginal microbiome provided new insights into its role in vaginal health, sexually transmitted infections (STIs), pregnancy, pregnancy outcomes, and gynecologic cancers (1–11). A healthy female reproductive tract is characterized by lactobacilli dominance, and up to 20 *Lactobacilliaceae* species have been reported in the lower female reproductive tract (12, 13). Five cervicovaginal microbiome community types have been described. Four are dominated by either Lactobacillus crispatus, Lactobacillus iners, Lactobacillus paragasseri (recently reclassified from Lactobacillus gasseri), or Lactobacillus mulieris (recently reclassified from Lactobacillus jensenii), and a fifth type is characterized by *Lactobacillus* depletion and high species diversity (13).

Lactobacilli are pivotal for cervicovaginal health and protect against pathogenic diseases, such as urinary tract infections, sexually transmitted infections, and bacterial vaginosis (BV) (14–19). The protective role of lactobacilli was first attributed to the production of lactic acid, which effectively lowers the local pH and generates an inhospitable environment for pathogenic bacteria (20–22). It is now understood that in addition to the production of lactic acid, vaginal lactobacilli employ multiple exclusionary mechanisms, such as bacteriocins, antimicrobial metabolites, stable colonization, and the heavily debated bacterial production of hydrogen peroxide (23–27).

Although L. crispatus, *L. iners*, *L. paragasseri*, and *L. mulieris* are frequently recovered from the cervicovaginal environment, L. crispatus and *L. iners* are the most prevalent of these species (25). In the context of vaginal pH modulation, L. crispatus is positively correlated with the lowest pH, L. jensenii and L. gasseri modulate pH at moderate levels, and *L. iners* is correlated with the highest pH (24). Relative to L. crispatus, *L. iners* less effectively prevents colonization of the cervicovaginal environment with bacterial vaginosis-associated bacteria (BVAB) and often coexists with other anaerobic bacteria (28). Thus, colonization with *L. iners* is often termed the “transition state,” as the dominance of this *Lactobacillus* species has been associated with the transition between health and disease and even observed as transitional between hormonal states, such as menses, or after antibiotic usage and sexual activity (29, 30). Among the four most common vaginal *Lactobacillus* species, L. gasseri is believed to be the most stable community over time (31, 32). L. gasseri has been also shown to inhibit pathogen adhesion to cervical cells (33). Similar to L. crispatus, L. jensenii exhibits protective features, such as immune modulation, antimicrobial properties, and competitive adherence to epithelial cells (24–26, 34, 35). Although L. jensenii and L. gasseri are common in the vaginal microenvironment, key strains of L. gasseri and L. jensenii that are frequently employed in vaginal microbiome research have recently been reclassified to *L. paragasseri* and *L. mulieris*, respectively, highlighting the importance of species- and strain-specific investigations (36, 37).

Lactobacilli produce postbiotics, broadly defined as bacterially produced compounds that confer beneficial properties, such as antibiofilm, antioxidant, pathogen-inhibitory, and immunomodulating activities (24). As such, these lactobacilli may be exploited for potential clinical applications and therapies; however, current vaginal probiotics are still undergoing testing and require effective formulation to support optimal gynecologic and reproductive health (38). Some hindrances could be the use of lactobacilli that do not normally dominate the vaginal microbiome and that lack the ability to colonize the environment in the long term (39). Clinically, lactobacilli strains vary in their ability to induce inflammatory cytokines (40), thus further supporting the need for immunometabolic studies leveraging metabolomic data sets. A vaginal L. crispatus isolate, Lactin-V, has been debated for its effectiveness in treating BV and decreasing the abundance of BVAB and whether this treatment is a viable option for all individuals suffering from BV (36–38). Vaginal L. crispatus and L. gasseri have been demonstrated to produce a milieu of immunoreactive proteins (39). Additional clinical studies have indicated that lactobacilli are positively correlated with lactate and 4-hydroxyphenylacetate, isoleucine, leucine, tryptophan, phenylalanine, aspartate, dimethylamine, sarcosine, and pi-methylhistidine, all of which are typically associated with vaginal health (40, 41). However, *in vitro* studies on the metabolic contributions of vaginal lactobacilli species in the context of human epithelial cells, the first responders to bacterial interaction, are limited. Therefore, our study aimed to provide further insight into the function of these vaginal lactobacilli and relate the findings to those observed in clinical settings.

We recently reported on the metabolomic and immunoproteomic characteristics of select BVAB and two L. crispatus strains by using our well-characterized organotypic three-dimensional (3D) cervical epithelial cell (CEC) model (42). In the current study, we utilized the 3D CEC model to extend previous findings and investigate the metabolic contributions of three common vaginal *Lactobacillus* strains: *L. paragasseri* JV-V03 (43–45) (formerly classified as L. gasseri JV-V03), *L. mulieris* JV-V16 (46, 47) (formerly classified as L. jensenii JV-V16), and *L. iners* AB-107 (30, 48–50). We focused on the cervix, as this body site is vulnerable to invading STI pathogens and dysbiotic microbiome sequelae, including preterm birth. Our 3D human CEC model accurately resembles ultrastructural features of cervical tissue *in vivo* and exerts physiologically relevant responses following colonization by various vaginal bacteria (51–55). By integrating our 3D CEC model and global untargeted metabolomics, we identified key metabolites related to epithelial homeostasis and lactobacilli colonization in the cervicovaginal niche. This study provides a foundation for future targeted investigations of these key metabolites and putative postbiotics that could be formulated for clinical modulation.

## RESULTS

### Vaginal lactobacilli exhibit close genomic similarity.

**FIG 1:**
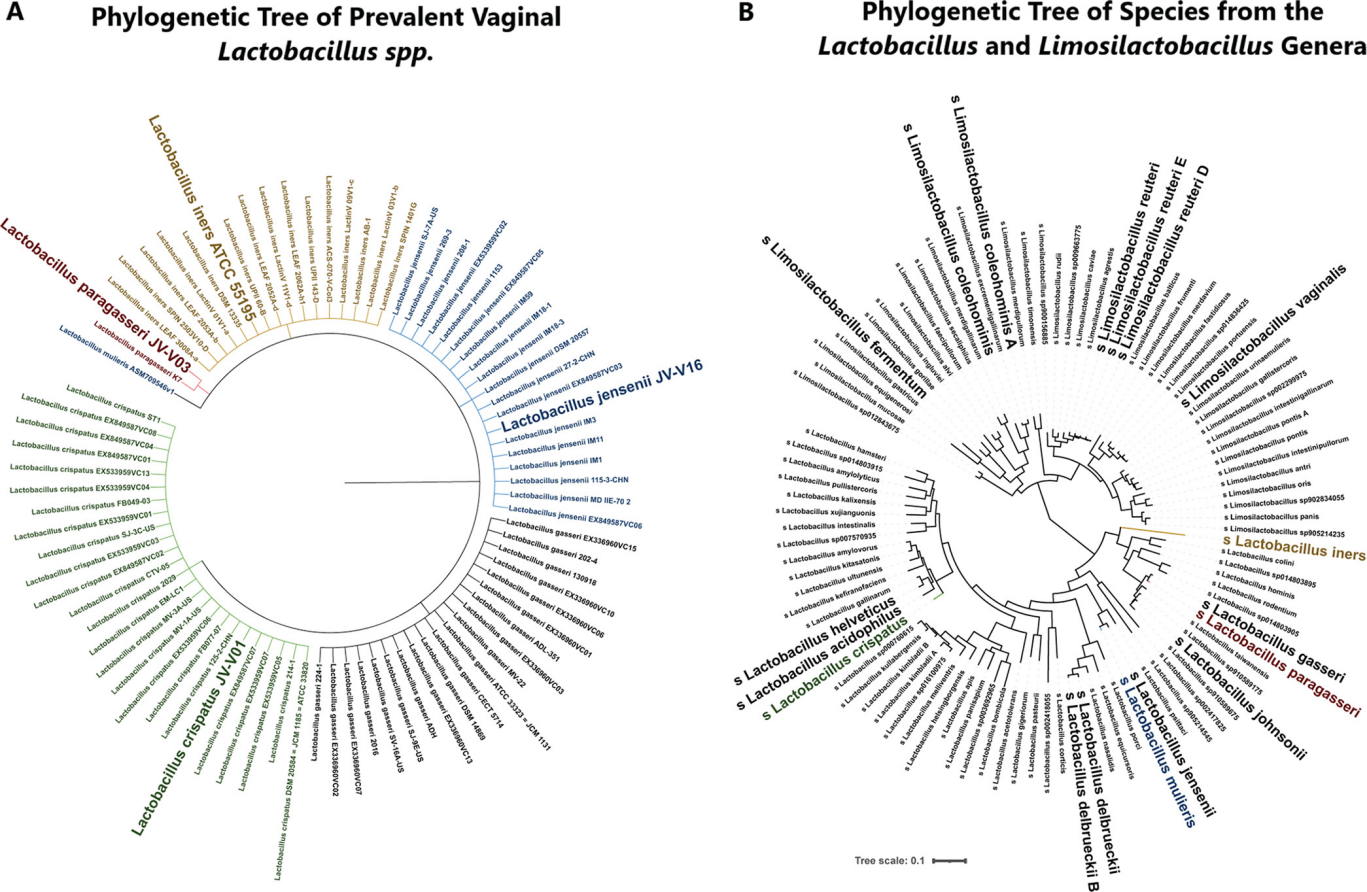
Taxonomic and phylogenetic trees of *Lactobacillus* and *Limosilactobacillus*, highlighting vaginally relevant species and recent taxonomic reclassifications. (A) Taxonomic tree constructed for genomes from the genus *Lactobacillus* in the NCBI repository and taxonomic lineages determined by NCBI’s Taxonomy browser, highlighting the most common vaginal lactobacilli species: *L. iners* (yellow), L. crispatus (green), L. jensenii and *L.mulieris* (blue), L. gasseri (black), and *L. paragasseri* (red). (B) Phylogenetic tree constructed from available genomes from NCBI and GTDB, where all strains are collapsed per species to better view lineages of all current species from both *Lactobacillus* and *Limosilactobacillus* genera. Species in bold indicate species that have been identified in vaginal samples from vaginal microbiome literature. The species utilized in the experimental study are in bold and colored: *L. iners* (yellow), *L. mulieris* (blue), and *L. paragasseri* (red).

Our study focused on three well-characterized vaginal *Lactobacillus* strains: *L. paragasseri* JV-V03, *L. mulieris* JV-V16, and *L. iners* AB-107 (Table 1) (44, 46, 56). All strains were obtained from either the American Type Culture Collection (ATCC) or the Biodefense and Emerging Infections Research Resources Repository (BEI Resources) and were isolated from the lower female reproductive tract. Genome sequences of these strains were then obtained from the National Center for Biotechnology Information (NCBI) GenBank. Two genomic trees, one constructed utilizing taxonomic lineages identified by NCBI and another constructed from phylogenetic relationships utilizing genomes from the Genome Taxonomy Database (GTDB). GTDB was utilized as it has the most up-to-date taxonomic classifications. These constructed trees validated previously established vaginal lactobacilli phylogeny (20, 57) (Fig. 1). *L. mulieris* had the closest relationship to L. jensenii but was also close taxonomically and phylogenetically to L. crispatus, compared to *L. iners* and *L. paragasseri* (Fig. 1). This phylogenetic relationship may explain the similar antimicrobial mechanisms employed by *L. mulieris* and L. crispatus (24, 58, 59). Other species that may be of interest to investigate further that have a close relationship with L. crispatus are L. helveticus, L. acidophilus, L. delbrueckii, and *L. delbrueckii* B. These species have also been investigated in the gut and vagina as beneficial probiotics (60–62).

**TABLE 1:** Genomic characteristics of three vaginal strains used in wet lab experiments

Characteristic	*L. mulieris*	*L. paragasseri*	*L. iners*
Strain	JV-V16	JV-V03	AB-107
Accession no.	ACGQ00000000.2	ACGO00000000.2	AEPX00000000.1
Isolation site	Female urogenital tract	Female urogenital tract	Vagina
Strain source	BEI	BEI	ATCC
Biorepository taxonomy	L. jensenii	L. gasseri	*L. iners*
NCBI taxonomy	L. jensenii	*L. paragasseri*	*L. iners*
GTDB taxonomy	*L. mulieris*	*L. paragasseri*	*L. iners*
No. of contigs	1	1	7
GC content (%)	34.39	34.81	32.55
No. of plasmids	0	0	0
Contig *L*_50_	1	1	1
Genome length (bp)	1,604,632	1,967,870	1,238,993
*N*_50_ (bp)	1,604,632	1,967,870	1,229,783
No. of CDSs	1,449	1,925	1,156
No. of tRNAs	55	59	48
No. of repeat regions	40	33	8
No. of rRNAs	10	9	3
No. of hypothetical proteins	299	470	201

In contrast, *L. paragasseri* had the closest phylogenetic relationship to L. gasseri and was observed to be closer taxonomically and phylogenetically to *L. iners* compared to L. crispatus or *L. mulieris* (Fig. 1). The only other lactobacilli known to colonize the vaginal environment with a close phylogenetic relationship to this group was L. johnsonii. *L. johnsonni* has been shown to have metabolic properties that may provide protection against Candida albicans (63, 64). These relationships provide a global picture of the variation of potential metabolic benefits of vaginal *Lactobacilliaceae* beyond L. crispatus.

**FIG 2:**
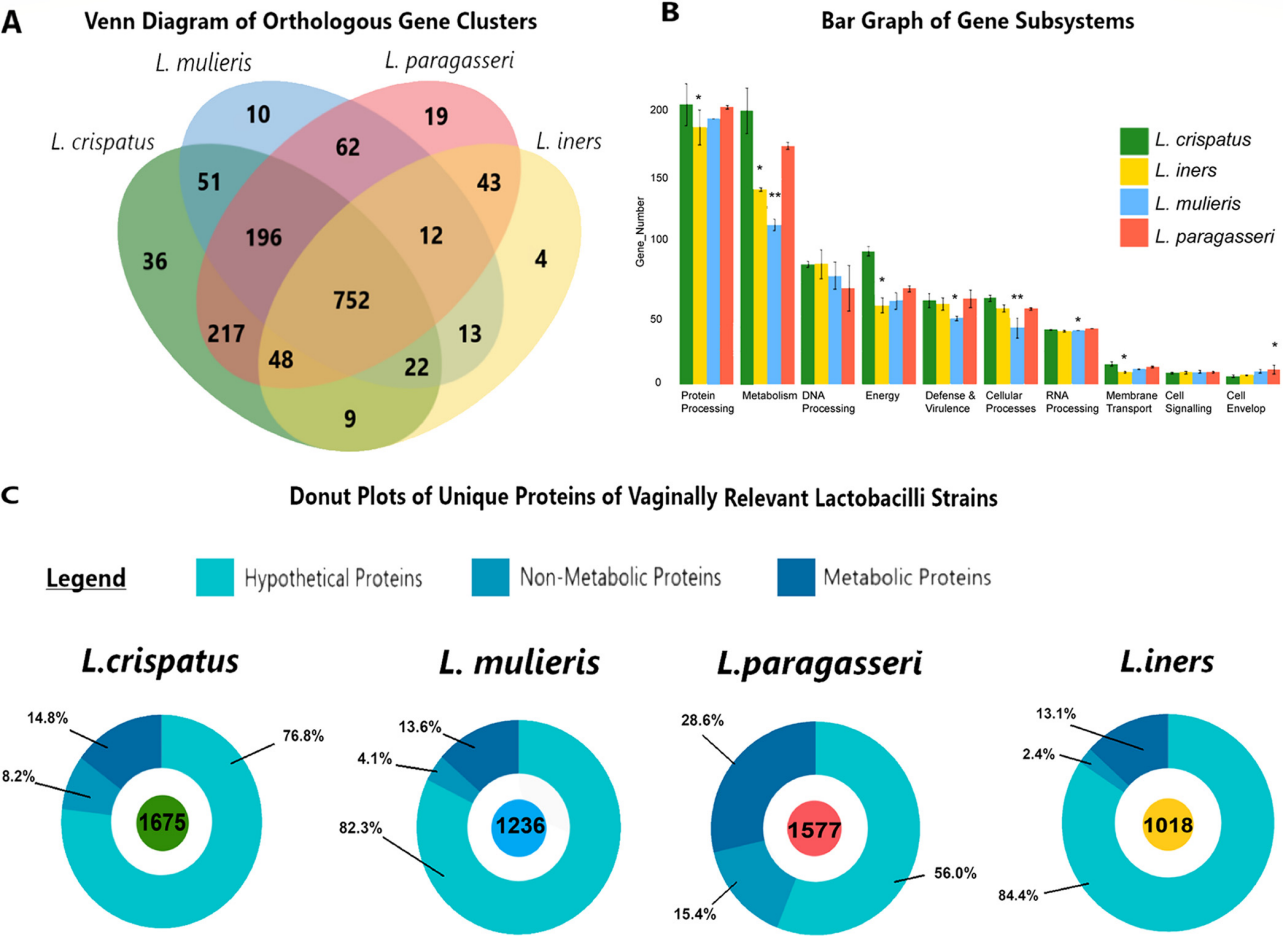
Orthologous gene clusters, annotated gene subsystems, and unique proteins between key vaginal lactobacilli. (A) Venn diagram, revealing gene clusters shared by the vaginal lactobacilli representatives: *L. iners* AB-107 (yellow), *L. paragasseri* JV-V03 (red), L. crispatus JV-V01 (green), and *L. mulieris* JV-V16 (blue). (B) Bar graph of gene subsystems, annotated by PATRIC from whole-genome sequences and experimental strain representatives. Kruskal-Wallis and Dunn’s tests were utilized to calculate significant differences. Pairwise comparisons in the image are relative to L. crispatus. *, *P < *0.05; **, *P < *0.01; ***, *P < *0.001; ****, *P < *0.0001. (C) Donut plots of predicted proteins identified by PATRIC to be unique to each *Lactobacillus* species. Proteins are categorized into the following: hypothetical proteins (light blue), nonmetabolic proteins (medium blue), and proteins related to metabolism (dark blue). Percentages of the putative protein categories are indicated in the panel.

L. crispatus JV-V01 was utilized as an additional comparator in genomic analyses, because similar metabolomic analyses were performed in a prior study by Laniewski et al. (42). Shared orthologous genes identified by OrthoVenn2 (65) confirmed that, overall, L. crispatus JV-V01, *L. iners* ATCC 55185 (AB-1 genome), *L. paragasseri* JV-V03, and *L. mulieris* JV-V16 strains were closely related and shared 752 orthologous genes (Fig. 2A). L. crispatus had the largest genome at 2.32 Mb, while *L. iners* had the smallest genome at 1.38 Mb (Table 1; see also Fig. S1 and Table S1 in the supplemental material). Similarly, GC content differed between L. crispatus (37.10%) and *L. iners* (33.12%). *L. mulieris* had a genome size of 1.63 Mb, and *L. paragasseri* was 1.96 Mb in length, and these two had GC contents of 34.43% and 34.91%, respectively (Table 1, Fig. S1, and Table S1). Low GC content and small genome size are attributed to host adaptation (57, 66, 67) and are observed in vaginal organisms, including vaginal lactobacilli. These differences were also reflected in the orthologous gene profiles, with L. crispatus encoding 1,331 genes (36 unique to the strain), *L. paragasseri* encoding 1,349 genes (19 unique to the strain), *L. mulieris* encoding 1,118 genes (10 unique), and *L. iners* encoding 903 genes (4 unique) (Fig. 2A). Genome annotation of the strains utilized in this study as well as additional whole genomes of strains of L. crispatus, *L. iners*, *L. paragasseri*, and *L. mulieris* were identified by PATRIC (68) and revealed that, overall, the highest average number of genes (197) participated in the subsystem of protein processing, and the lowest number (9) of genes were dedicated to the cellular envelope (Fig. 2B and Table S2). Species-specific differences were exemplified, with L. crispatus and *L. paragasseri* having a higher average number of metabolism-related genes (200 and 175, respectively) than *L. iners* and *L. mulieris* (143 and 117, respectively). However, significant differences were only observed between L. crispatus versus *L. iners* (*P = *0.002) and L. crispatus versus *L. mulieris* (*P < *0.001) (Fig. 2B and Table S2). Intriguingly, multiple-strain analysis revealed that L. crispatus codes for a statistically higher average number of hypothetical proteins (675) compared to *L. iners* (289) and *L. mulieris* (320) (*P = *0.004 and *P = *0.033, respectively) (Fig. 2B and Table S2). The PATRIC protein sorter (68) also identified putative proteins unique to strains: L. crispatus JV-V01 (627), *L. iners* AB-107 (327), *L. mulieris* JV-V16 (566), and *L. paragasseri* JV-V03 (241). Most strains encoded species-specific unique hypothetical proteins (Fig. 2C). Additionally, *L. paragasseri* had the highest percentage of unique putative metabolic proteins, 28.6% (69/241). In comparison, the other lactobacilli strains had similar percentages of unique metabolic proteins: 14.8% (93/627) for L. crispatus JV-V01, 13.1% (43/327) for *L. iners* AB-107, and 13.6% for *L. mulieris* JV-V16 (77/566). L. crispatus had the most nonmetabolic proteins, 8.2% (52/627), with the majority functioning as defense or stress proteins, which may be beneficial for homeostasis in the cervicovaginal environment. Genomic analyses verified that *L. paragasseri* and *L. mulieris* are genetically more similar to L. crispatus, whereas *L. iners* significantly differed from other vaginal lactobacilli.

10.1128/msphere.00452-22.1 TABLE S1 Genomic characteristic differences between lactobacilli. This table contains overall genomic characteristics (GC content, genome size, coding sequences, proteins with known functions, and hypothetical proteins) and the differences among 26 strains of four *Lactobacillus* species: L. crispatus, *L. iners*, L. jensenii or *L. mulieris*, and *L. paragasseri* strains were obtained from the GenBank database as of 1 October 2021. Download Table S1, XLSX file, 0.01 MB. Copyright © 2023 Jimenez et al. 2023 Jimenez et al. https://creativecommons.org/licenses/by/4.0/ This content is distributed under the terms of the Creative Commons Attribution 4.0 International license.

10.1128/msphere.00452-22.2 TABLE S2 Predicted gene subsystem differences between lactobacilli. This table contains overall annotated genes of 26 lactobacilli strains from L. crispatus*, L. iners*, L. jensenii or *L. mulieris*, and *L. paragasseri* that were categorized into subsystems by PATRIC. The categories were protein processing, metabolism, DNA processing, defense and virulence, RNA processing, energy, cellular processes, membrane transport, cell signaling, and cell transport. Download Table S2, XLSX file, 0.01 MB. Copyright © 2023 Jimenez et al. 2023 Jimenez et al. https://creativecommons.org/licenses/by/4.0/ This content is distributed under the terms of the Creative Commons Attribution 4.0 International license.

10.1128/msphere.00452-22.6 FIG S1 Genomic characteristics of key vaginal lactobacilli. Differences in genomic characteristics of four well-characterized vaginal lactobacilli genomes are summarized. Numbers of coding DNA sequences (CDS), number of known putative proteins, and number of hypothetical proteins observed are shown in the top row. GC content in percentage and genome size in megabases observed are in the bottom row. Circularized whole-genome sequences and sequences from experimental strains of the vaginally relevant *Lactobacillus* species are in the following colors: *L. iners* (yellow), *L. paragasseri* (red), L. crispatus (green), and *L. mulieris* (blue). Statistical analyses were performed using the Kruskal-Wallis rank-sum test. *, *P < *0.05; **, *P < *0.01; ***, *P < *0.001; ****, *P < *0.0001. Download FIG S1, TIF file, 1.0 MB. Copyright © 2023 Jimenez et al. 2023 Jimenez et al. https://creativecommons.org/licenses/by/4.0/ This content is distributed under the terms of the Creative Commons Attribution 4.0 International license.

### Vaginal lactobacilli colonized the 3D CEC model similarly.

**FIG 3:**
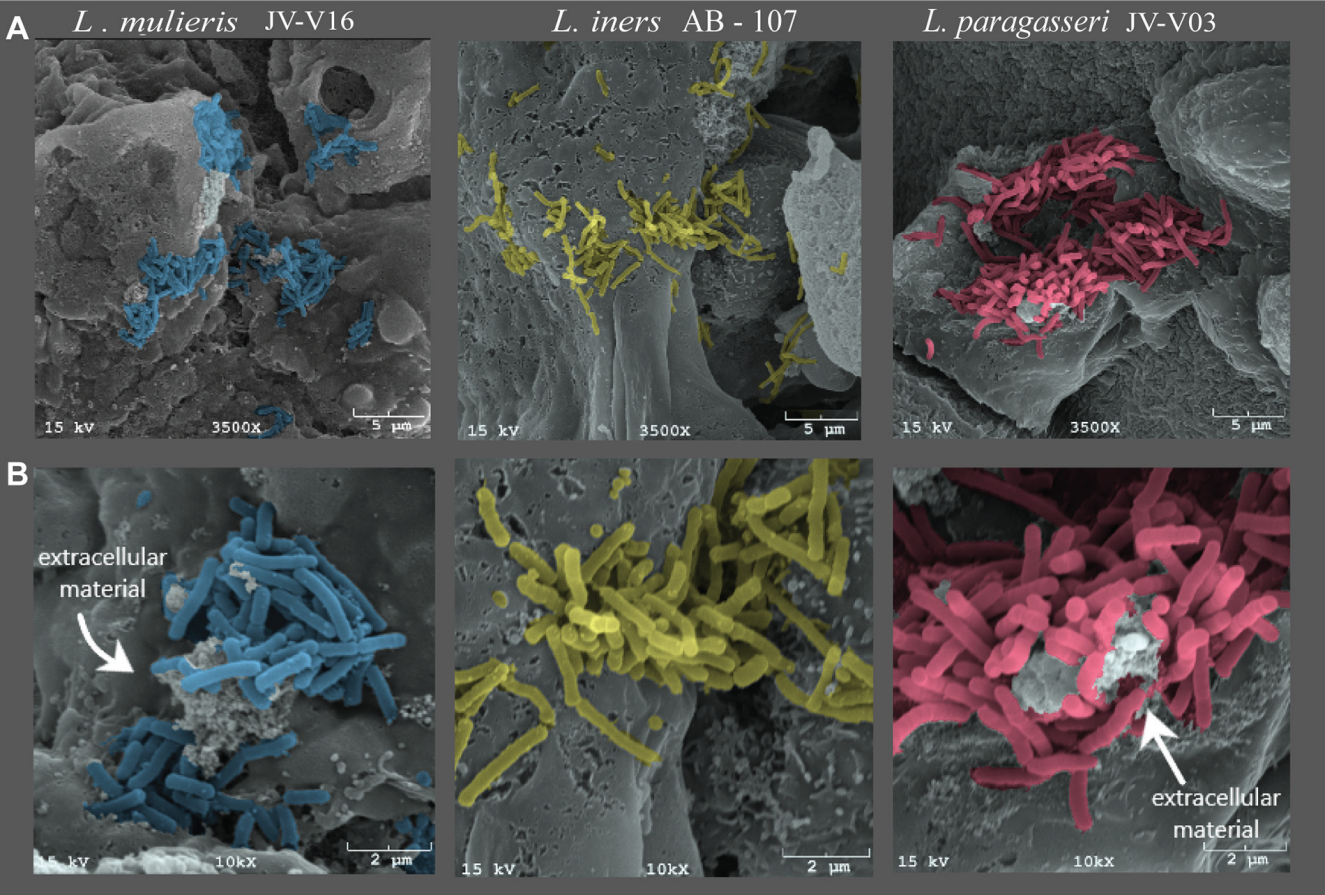
*L. mulieris* JV-V16, *L. iners* AB-107, and *L. paragasseri* JV-V03 colonize 3D human CEC models. Pseudocolored SEM images of *L. mulieris* JV-V16 (blue), *L. iners* AB-107 (yellow), and *L. paragasseri* JV-V03 (red) demonstrate colonization of 3D CECs. SEM images were captured at 3,500× (top) or 10,000× (bottom) magnification. All vaginal *Lactobacillus* strains exhibited a bacillus or rod-shaped morphology and colonized 3D CECs in aggregates. CECs colonized by *L. iners* AB-107 showed signs of cell stress. Arrows indicate extracellular material and bacterial cells that were regularly observed in the lactobacilli autoaggregates.

Human 3D CEC models were generated as previously described (53–55). These model systems are valuable for investigating host-microbe interactions as apical and basal polarity and tissue-like cellular organization are recapitulated. To confirm that vaginal lactobacilli strains effectively colonized our 3D CEC model, we performed scanning electron microscopy (SEM). The SEM analysis revealed that *L. paragasseri*, *L. mulieris*, and *L. iners* colonized 3D CEC models, usually in crevices and folds of the epithelial cells (Fig. 3). Lactobacilli strains were visualized as rod-shaped bacteria and exhibited autoaggregation in all bacterial inoculations of CEC. Autoaggregation is mediated by proteins (autoagglutinins) and exopolysaccharides on the bacterial cell surface that contribute to bacterial cell adhesion and protection from environmental stresses (69, 70). Extracellular material was also present in multiple SEM micrographs of *L. paragasseri* and *L. mulieris* inoculations, and this may provide additional evidence of formation of a protective autoaggregative biofilm. In contrast, 3D CEC inoculated with *L. iners* displayed cell blebbing and signs of CEC membrane disruption in multiple SEM micrographs of the CEC inoculated models.

To investigate the CEC immune response to vaginal *Lactobacillus* species, we performed a multiplex immunoassay which quantified secreted cytokines (interleukin 1α [IL-1α], IL-1β, IL-1 receptor agonist [IL-1RA], IL-6, and tumor necrosis factor alpha [TNF-α]), chemokines (fractalkine, IL-8, interferon gamma-inducible protein 10 kDa [IP-10], monocyte chemoattractant protein 1 [MCP-1], MCP-3, macrophage inflammatory protein 1β [MIP-1β], and regulated on activation, normal T-cell expressed and secreted [RANTES]), and growth factors (platelet-derived growth factor [PDGF-AA], transforming growth factor alpha [TGF-α], and vascular endothelial growth factor [VEGF]). Cell culture supernatant protein concentrations from colonized 3D CEC were compared to those in phosphate-buffered saline (PBS) controls. In addition, we used the BVAB *Lancefieldella parvula* as a positive inflammatory control (42). Inoculation of 3D CEC revealed no significant differences in inflammatory chemokine, cytokine, or growth factor profiles (Fig. S2 and Table S4). Taken together, *L. parvula* induced a proinflammatory response (IL-8, MCP-1, and TGF-α) in 3D CEC to a greater extent than vaginal lactobacilli or the PBS mock-inoculated controls (Fig. S2 and Table S4).

10.1128/msphere.00452-22.4 TABLE S4 Student *t* test results for each individual strain (*L. iners* AB-107, *L. mulieris* JV-V16, and *L. paragasseri* JV-V03) compared to PBS mock-inoculated controls. This table contains statistical *t* test data from metabolomic analysis of *L. iners* AB-107 versus PBS controls, *L. mulieris* JV-V16 versus PBS controls, and *L. paragasseri* JV-V03 versus PBS controls. Download Table S4, XLSX file, 0.02 MB. Copyright © 2023 Jimenez et al. 2023 Jimenez et al. https://creativecommons.org/licenses/by/4.0/ This content is distributed under the terms of the Creative Commons Attribution 4.0 International license.

10.1128/msphere.00452-22.7 FIG S2 *L. parvula* significantly elevated production of inflammatory protein biomarkers compared to vaginal lactobacilli and PBS mock-inoculated controls in 3D CEC aggregates. Bio-Plex analysis was performed for cytokines, chemokines, and growth factors secreted by 3D human CECs infected with *L. iners* AB-107, *L. mulieris* JV-V16, and *L. paragasseri* JV-V03 for 24 h under anaerobic conditions. TGF-α, IL-8, and MCP-1 were all significantly elevated by *L. parvula*, while all lactobacilli exhibited no notable change to PBS controls. One-way ANOVA and Bonferroni *post hoc* multiple comparisons determined statistical significance. *, *P < *0.05; **, *P < *0.01; ***, P < 0.001; ****, *P < *0.0001. Download FIG S2, TIF file, 1.4 MB. Copyright © 2023 Jimenez et al. 2023 Jimenez et al. https://creativecommons.org/licenses/by/4.0/ This content is distributed under the terms of the Creative Commons Attribution 4.0 International license.

### Metabolic profiles of host vaginal lactobacilli are functionally different between species.

**FIG 4:**
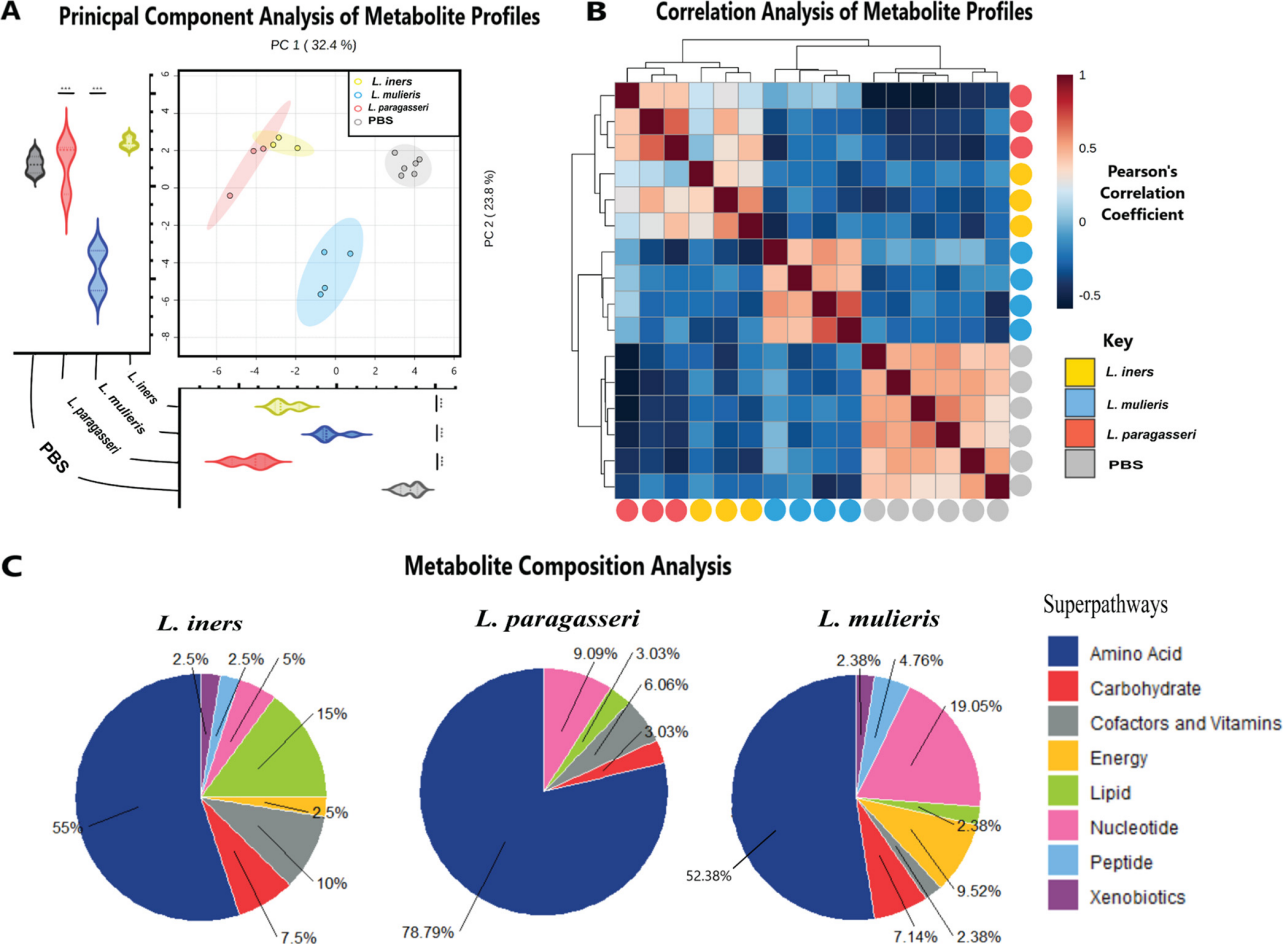
Distinct metabolic profiles of *L. mulieris* JV-V16, *L. paragasseri* JV-V03, and *L. iners* AB-107. (A) PCA of metabolic profiles showed moderate clustering between *L. iners* AB-107, *L. mulieris* JV-V16, *L. paragasseri* JV-V03, and the PBS mock-inoculated controls. PC1 and PC2 scores were statistically analyzed by one-way ANOVA with Bonferroni *post hoc* tests. *, *P < *0.05; *, *P < *0.01; ***, *P < *0.001; ****, *P < *0.0001. (B) Heatmap of Pearson correlation coefficients of metabolic profiles. Clustering was performed using Euclidean distance and Ward linkage of biological replicates for each experimental group. (C) Pie charts of percentages of significantly (*P < *0.05) altered metabolites determined by unpaired Student's *t* test, grouped by superpathway, and compared to PBS mock-infected controls (total number of significantly changed metabolites for *L. iners* AB-107, *L. mulieris* JV-V16, and *L. paragasseri* JV-V03 were 40, 42, and 33, respectively). No significant differences were observed between composition of superpathways as determined by chi-squared test.

To decipher how *L. paragasseri*, *L. mulieris*, and *L. iners* modulate the extracellular cervicovaginal microenvironment’s metabolome, we performed global untargeted metabolomics using supernatants collected from 3D CEC experiments. Untargeted metabolomics identified 314 metabolites, of which 103 were significant based on analysis of variance (ANOVA) between the four experimental groups. We utilized principal-component analysis (PCA) and Pearson’s correlation analysis to compare global metabolomic profiles between each species (Fig. 4). Biological replicates from each bacterial condition and PBS controls were distinctly clustered by PCA, although some overlap of *L. paragasseri* and *L. iners* metabolic profiles was identified (Fig. 4A). Principal component 1 (PC1) explained 32.4% of the variance, while principal component 2 (PC2) explained 23.8% of the variance scores. PC1 was significantly different (*P < *0.001) between all *Lactobacillus* species and PBS-inoculated controls; PC2 contributed to significant differences (*P < *0.001) between *L. paragasseri* versus PBS-inoculated controls and *L. mulieris* versus PBS-inoculated controls. Pearson’s correlation analysis demonstrated that for each biological replicate the metabolic profile of the different *Lactobacillus* species and the PBS-inoculated controls were grouped by species (Fig. 4B). Similar to our genomic findings, *L. iners* and *L. paragasseri* exhibited similar metabolic profiles.

To better understand the overall differences of the specific metabolites among tested lactobacilli, we performed Student’s *t* tests with Welch’s correction and the false-discovery rate (FDR) to compare each *Lactobacillus* species to the PBS-inoculated controls. Overall, *L. paragasseri*, *L. mulieris*, and *L. iners* significantly (*P < *0.05) altered 33, 42, and 40 metabolites, respectively, compared to PBS-inoculated controls (Table S3). Of these, all *Lactobacillus* species shared 7/54 significantly altered metabolites: alpha-hydroxyisocaproate, 1-methyladenine, 3-(4-hydroxyphenyl) lactate (HPLA), cytosine, threonine, tryptophan, and ribulose and xylulose (Table S3, Fig. S3). *L. paragasseri*, *L. mulieris*, and *L. iners* uniquely altered 11, 18, and 19 metabolites, respectively (Table S3, Fig. S3). *L. paragasseri* and *L. iners* shared four significantly altered metabolites specific to these bacterial inoculations (Table S3).

10.1128/msphere.00452-22.3 TABLE S3 Immunoproteomic differences between *L. iners* AB-107, *L. mulieris* JV-V16, *L. paragasseri* JV-V03, *L. parvula* DNF00906, and PBS control. This table contains immunoproteomic data of *L. iners* AB-107, *L. mulieris* JV-V16, *L. paragasseri* JV-V03, *L. parvula* DNF00906, and PBS controls. The cytokines and chemokines that were observed were IL-1α, IL-1β, IL-RA, IL-6, IL-8, IP-10, fractalkine, MCP-1, MCP-3, MIP-1β, PDGF-AA, RANTES, TGF-α, TNF-α, and VEGF. Download Table S3, XLSX file, 0.01 MB. Copyright © 2023 Jimenez et al. 2023 Jimenez et al. https://creativecommons.org/licenses/by/4.0/ This content is distributed under the terms of the Creative Commons Attribution 4.0 International license.

Next, we categorized all significantly altered metabolites by superpathway (amino acid, carbohydrate, cofactors and vitamins, energy, lipid, nucleotide, peptide, and xenobiotics). Metabolites representing the amino acid superpathway were highly altered by all lactobacilli: *L. paragasseri* (78.79%), *L. mulieris* (52.38%), and *L. iners* (55.0%) (Fig. 4C). Metabolites related to the lipid superpathway (3.03%, 2.38%, and 15.0%) and cofactors and vitamins superpathway (6.06%, 2.38%, and 10%) were more abundant in *L. iners* than in other lactobacilli. In contrast, the nucleotide superpathway (9.09%, 19.05%, and 5.0%) and energy superpathway (0.0%, 9.52%, and 2.5%) were more abundant in *L. mulieris* than in other lactobacilli. The compositions of the remaining superpathways were not significantly different between the tested *Lactobacillus* species, potentially indicating a large overlap of function among the vaginal lactobacilli.

**FIG 5:**
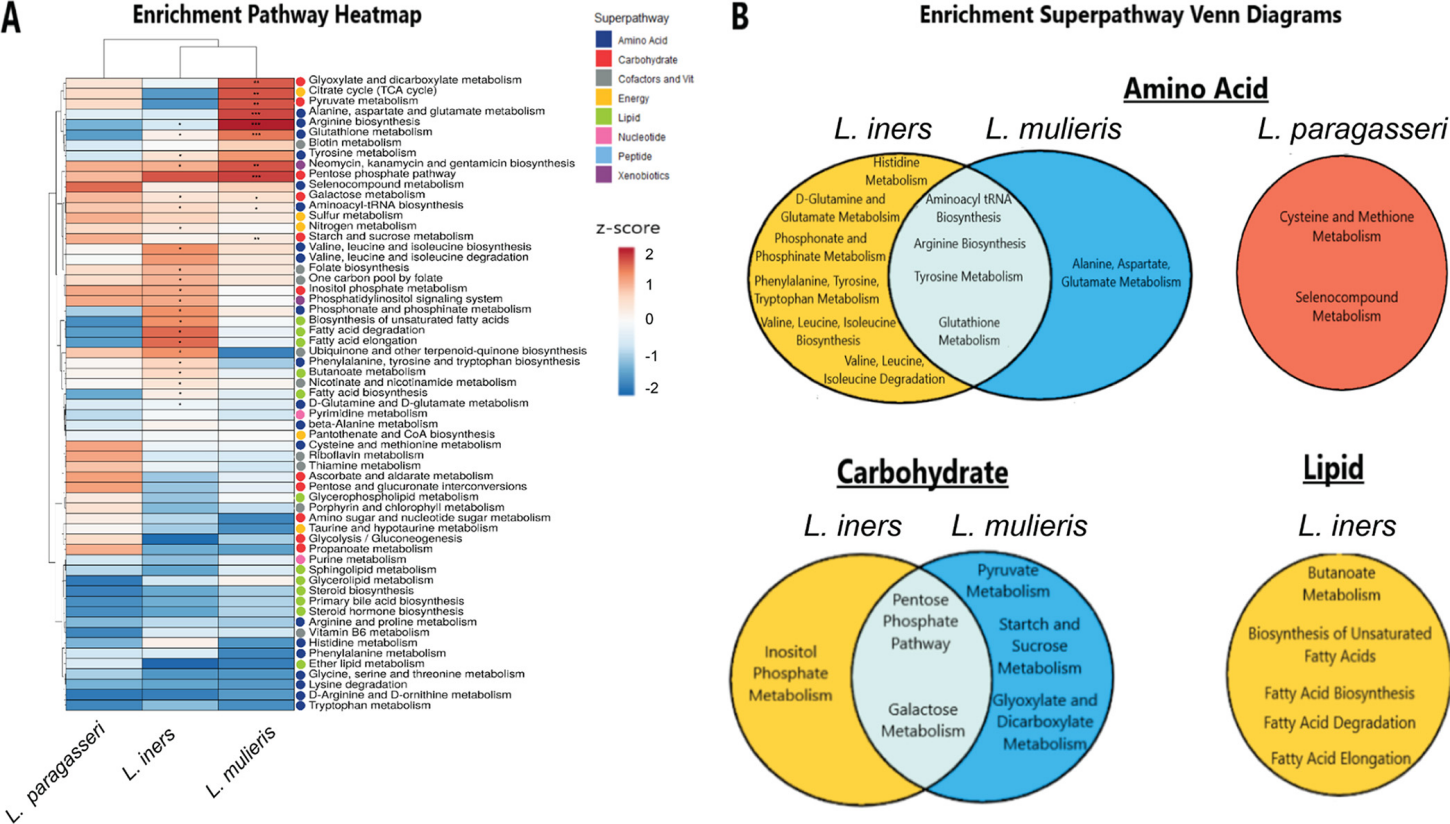
Vaginal lactobacilli metabolic enrichment analysis indicated alterations of amino acid, carbohydrate, and lipid subpathways. (A) Heatmap of metabolic pathway enrichment clustered by Euclidean distance and Ward linkage for *L. iners* AB-107, *L. mulieris* JV-V16, and *L. paragasseri* JV-V03. Subpathways that were significantly enriched (*P < *0.05) in metabolite set enrichment analysis are indicated within heatmap cells. *, *P < *0.05; ***, *P < *0.001; ****, *P < *0.0001. Colored circles indicate the superpathway to which the subpathways are linked. (B) Venn diagrams comparing the most common significantly altered metabolites categorized by superpathway (amino acids, carbohydrates, and lipids) compared to PBS mock-inoculated controls by Student's *t* test (*P < *0.05).

To examine alterations of metabolic pathways and their differences among vaginal lactobacilli, we conducted metabolic pathway enrichment analysis with KEGG pathways to identify metabolic pathways that were significantly enriched and the corresponding metabolites that were differentially modulated by each *Lactobacillus* species (Fig. 5). This analysis revealed significantly (*P < *0.05) enriched pathways from all lactobacilli. There were 25, 13, and 2 subpathways significantly enriched by *L. iners*, *L. mulieris*, and *L. paragasseri*, respectively (Fig. 5A and Table S5). *L. paragasseri* exhibited significantly enriched cysteine and methionine metabolism (enrichment score = 82.77; *P = *0.039) and selenocompound metabolism (enrichment score = 94.249; *P = *0.039), which participate in amino acid metabolism (Fig. 5A). Most metabolic pathways significantly enriched by *L. mulieris* belonged to amino acid and carbohydrate pathways, with arginine biosynthesis (enrichment score = 96.11; *P < *0.001) being the most highly enriched (Fig. 5A and Table S5). *L. iners* altered subpathways in amino acid and lipid metabolism; however, the most highly enriched subpathway was the pentose phosphate pathway, which belongs to carbohydrate metabolism (enrichment score = 95.93; *P = *0.016) (Fig. 5A and Table S5).

10.1128/msphere.00452-22.5 TABLE S5 Metabolite set enrichment analysis significant results for each individual strain (*L. iners* AB-107, *L. mulieris* JV-V16, and *L. paragasseri* JV-V03) compared to PBS mock-inoculated controls. This table reports significant (*P < *0.05) statistical enrichment data from metabolomic analysis of *L. iners* AB-107 versus PBS controls, *L. mulieris* JV-V16 versus PBS controls, and *L. paragasseri* JV-V03 versus PBS controls. Download Table S5, XLSX file, 0.01 MB. Copyright © 2023 Jimenez et al. 2023 Jimenez et al. https://creativecommons.org/licenses/by/4.0/ This content is distributed under the terms of the Creative Commons Attribution 4.0 International license.

10.1128/msphere.00452-22.8 FIG S3 Vaginal *Lactobacillus* species had significantly differentiated metabolic profiles from mock-inoculated controls. (A) Stacked bar chart of subpathways of significant metabolites identified by Student’s *t* tests with Welch’s correction and compared to PBS mock-inoculated controls. (B) Venn diagram indicating the unique and overlapping metabolites that were significantly altered (*P < *0.05) between the lactobacilli inoculations. Download FIG S3, TIF file, 1.2 MB. Copyright © 2023 Jimenez et al. 2023 Jimenez et al. https://creativecommons.org/licenses/by/4.0/ This content is distributed under the terms of the Creative Commons Attribution 4.0 International license.

10.1128/msphere.00452-22.9 FIG S4 Venn diagrams of remaining metabolic superpathways enriched in 3D CEC models inoculated with *L. iners*, *L. mulieris*, and *L. paragasseri*. Metabolite pathway enrichment analysis results are shown for human 3D CEC models inoculated with vaginal lactobacilli for 24 h at 37°C under anaerobic conditions. Venn diagrams with the remaining superpathways from enrichment pathway analysis are also shown. Only *L. iners* and *L. mulieris* had significantly enriched pathways for the remaining pathways: the vitamins and cofactors pathway and the xenobiotics and energy pathway. Download FIG S4, TIF file, 1.7 MB. Copyright © 2023 Jimenez et al. 2023 Jimenez et al. https://creativecommons.org/licenses/by/4.0/ This content is distributed under the terms of the Creative Commons Attribution 4.0 International license.

We next compared the relatedness between superpathway enrichment levels among the lactobacilli. Amino acid, carbohydrate, and lipid superpathways were the most prevalent enriched pathways (Fig. 5B and Table S5). Significant (*P < *0.05) enrichment of metabolic subpathways belonging to the amino acid superpathway were prevalent among all lactobacilli (Fig. 5B). *L. mulieris* and *L. iners* were the only species that enriched carbohydrate metabolic pathways (Fig. 5B). Additionally, *L. iners* uniquely enriched subpathways participating in lipid metabolism (Fig. 5B). *L. paragasseri* did not share significantly enriched subpathways with *L. iners* or *L. mulieris*. *L. iners* and *L. mulieris* shared eight pathways: four in amino acid, two in carbohydrate, one in cofactors and vitamins (nicotinate and nicotinamide metabolism), and one in xenobiotics (neomycin, kanamycin, and gentamicin biosynthesis) (Fig. 5B, Fig. S4, and Table S5).

**FIG 6:**
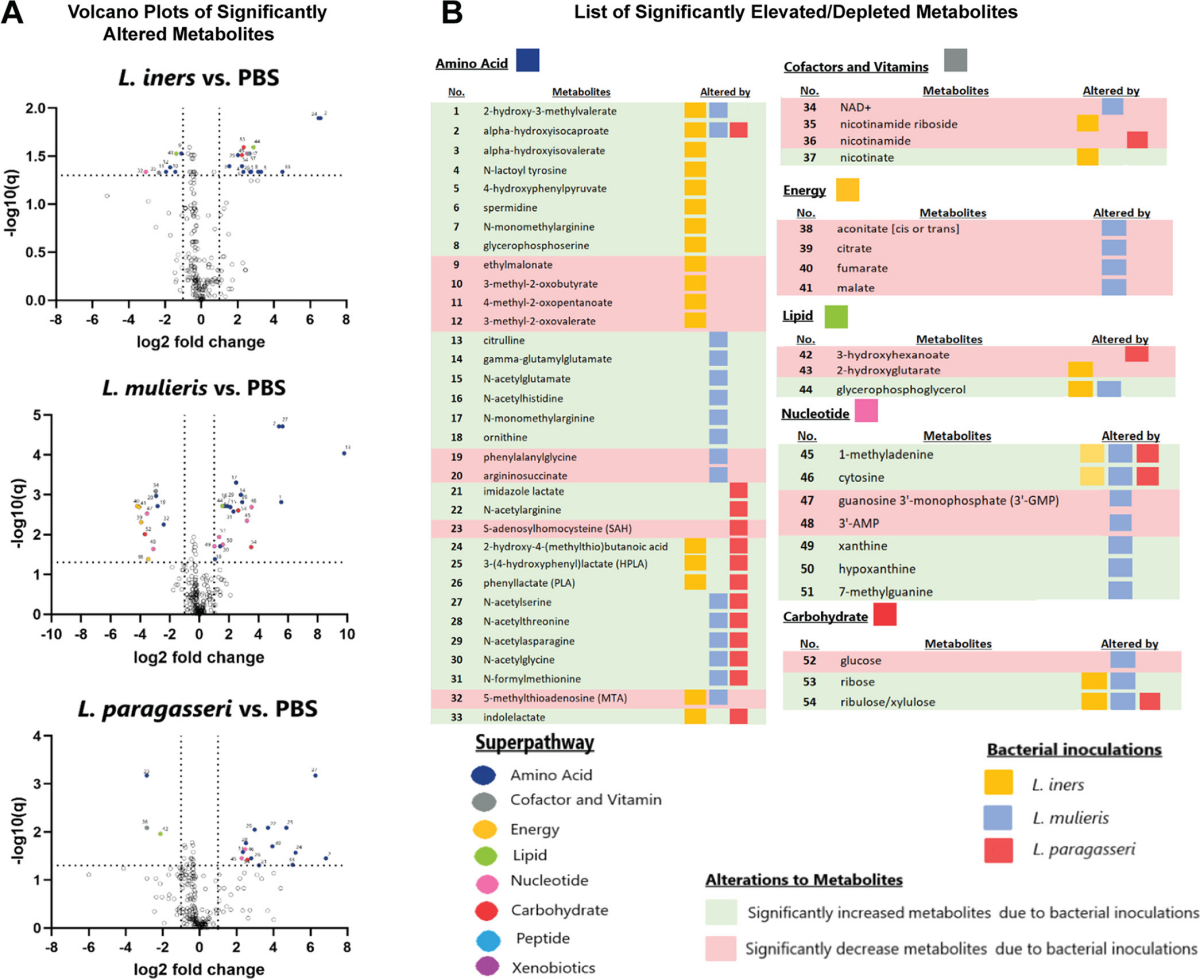
Vaginal *Lactobacillus* species had significant log-fold changes in metabolites related to amino acids, carbohydrates, and lipids. Human 3D CECs were inoculated with *L. paragasseri*, *L. mulieris*, or *L. iners* under anaerobic conditions for 24 h. (A) Volcano plots of *L. paragasseri*, *L. mulieris*, and *L. iners* metabolomic profiles. The *x* axis represents the log_2_ fold change values of scaled metabolite intensity values between the bacteria-inoculated samples and PBS mock-inoculated controls. The *y* axis represents the −log_10_(*q*) value of differential metabolite abundances between bacteria-inoculated samples and PBS mock-inoculated controls using an unpaired two-tailed Student's *t* test. (B) Significantly altered metabolites (*Q* < 0.05) with a fold change less than −1.5 or greater than 1.5 are colored by superpathway.

We next sought to identify specific metabolites that were significantly (*P < *0.05) depleted or accumulated in the extracellular supernatant after inoculation of a 3D CEC with *L. paragasseri*, *L. mulieris*, and *L. iners* relative to PBS controls (Fig. 6A). CECs inoculated with *L. paragasseri*, *L. mulieris*, or *L. iners* contained significantly elevated metabolites (15, 20, and 18, respectively), while other metabolites were significantly depleted (3 with *L. paragasseri*; 11 with *L. mulieris*; and 7 with *L. iners*). CECs inoculated by all three of these lactobacilli species had respective elevated levels of the following metabolites: alpha-hydroxyisocaproate (*P = *0.035, *P < *0.001, *P = *0.013), 1-methyladenine (*P = *0.035, *P = *0.004, *P = *0.03), cytosine (*P = *0.023, *P = *0.002, *P = *0.03), and ribulose and xylulose (*P = *0.038, *P = *0.021, *P = *0.031). *L. paragasseri* and *L. mulieris* in each of their respective CEC inoculations altered five of the same metabolites, and notably, all were conjugated amino acids: *N*-acetylserine (*P < *0.001, *P < *0.001), *N*-acetylthreonine (*P = *0.017, *P = *0.002), *N*-acetylasparagine (*P = *0.009, *P = *0.002), *N*-acetylglycine (*P = *0.02, *P = *0.02), and *N*-formylmethionine (*P = *0.026, *P = *0.003). *L. paragasseri* and *L. iners* shared four metabolites also related to conjugated amino acid metabolites: 2-hydroxy-4-(methylthio) butanoic acid (*P = *0.027, *P = *0.013), HPLA (*P = *0.008, *P = *0.031), phenyllactate (PLA) (*P = *0.035, *P = *0.046), and indolelactate (ILA) (*P = *0.048, *P = *0.046). *L. mulieris* and *L. iners* shared three elevated metabolites: 2-hydroxy-3-methylvalerate (*P* = 0.002, *P = *0.046), glycerophosphoglycerol (*P = *0.002, *P = *0.026), and ribose (*P = *0.002, *P = *0.026), and one depleted metabolite, 5-methylthioadenosine (MTA) (*P = *0.006, *P = *0.046). *L. mulieris* uniquely elevated six and depleted nine metabolites; five were amino acid derivatives and eight were involved in the citric acid cycle (Fig. 6B). *L. iners* uniquely elevated seven metabolites and depleted five metabolites; 10 were involved in amino acid metabolism and four in lipid metabolism (Fig. 6B). *L. paragasseri* uniquely elevated two amino acid metabolites and depleted two, a cofactor and lipid metabolite (Fig. 6B). Overall, 3D CEC models inoculated with lactobacilli induced significant alterations of metabolites associated with amino acid pathways (Fig. 6). We observed a depletion of cofactors and vitamins, such as nicotinamide derivatives, which are important cofactors in many enzymatic reactions. *L. mulieris* uniquely modulated metabolites that participate in the tricarboxylic acid cycle. We previously reported comparable results when 3D CEC were inoculated with L. crispatus (42). These analyses support the importance of amino acid and energy metabolism in vaginal lactobacilli metabolism.

### Utilization of amino acids and elevation of conjugated amino acids indicated a unique feature of vaginal lactobacilli.

**FIG 7:**
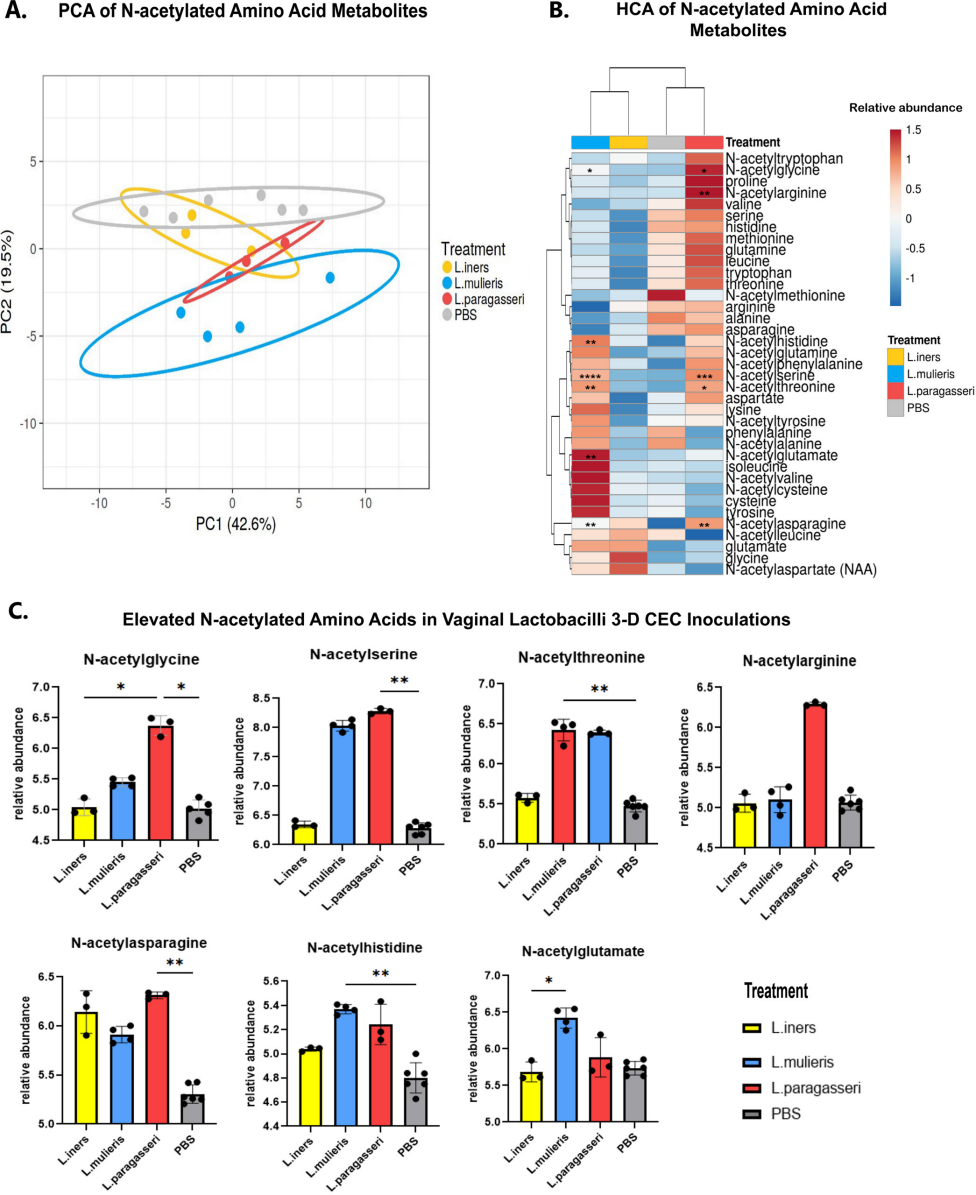
Vaginal *Lactobacillus* species alter amino acid metabolites. (A) PCA of amino acid and *N*-acetylated amino acid metabolic profiles indicated distinct clusters between *L. mulieris* JV-V16, *L. iners* AB-107, *L. paragasseri* JV-V03, and PBS mock-inoculated controls. (B) Heatmap of amino acid and *N*-acetylated amino acid metabolite relative abundance levels (means of biological replicates). Hierarchical clustering was performed using Euclidean distance and Ward linkage. Asterisks indicate significantly altered metabolites compared by unpaired Student's *t* test of each *Lactobacillu*s species versus PBS controls: *, *P < *0.05; **, *P < *0.01; ***, *P < *0.001; ****, *P < *0.0001. (C) Boxplots of *N*-acetylated amino acids that were significantly altered in the log-fold change analysis compared to PBS mock-inoculated controls, *L. iners* AB-107, *L. mulieris* JV-V16, and *L. paragasseri* JV-V03. Differences were observed between *N*-acetylated amino acids among all lactobacilli and PBS controls based on ANOVA (*P < *0.05) and adjusted for multiple tests by FDR. Significance is indicated by pairwise comparisons; *, *P < *0.05; **, *P < *0.01; ***, *P < *0.001; ****, *P < *0.0001.

To further elucidate amino acid metabolism in vaginal lactobacilli, we performed subanalyses on two main groups of conjugated amino acid metabolites. *N-*Acetyl amino acids were the most common amino acid metabolites that were altered by the vaginal lactobacilli. An additional PCA with only the proteinogenic amino acids and their *N*-acetylated derivatives revealed that the metabolic profiles in PC1, which accounted for 42.6% of the variance (*P = *0.813), had great overlap between each species and PBS controls. Only *L. mulieris* was significantly distinguishable by PC2, which accounted for 19.2% variance (*P < *0.001) (Fig. 7A). *L. iners* and *L. paragasseri* again exhibited a close metabolic relationship within this subset of metabolites. While hierarchical clustering revealed that, despite the lactobacilli sharing pathways in amino acid metabolism, they differed in terms of the relative abundance of specific amino acids and their *N*-acetylated derivatives (Fig. 7B). No proteinogenic amino acids significantly differed among the lactobacilli in our log-fold change analysis (Fig. 6A). Relative to PBS controls, *L. paragasseri* significantly altered *N*-acetylated amino acid derivatives: *N*-acetylglycine (*P = *0.02), *N*-acetylarginine (*P = *0.008), *N*-acetylserine (*P < *0.001), *N*-acetylthreonine (*P = *0.017), and *N*-acetylasparagine (*P = *0.009). *L. mulieris* similarly elevated *N*-acetylated amino acids: *N*-acetylglycine (*P = *0.02), *N*-acetylhistidine (*P = *0.002), *N*-acetylserine (*P < *0.001), *N*-acetylthreonine (*P = *0.002), *N*-acetylglutamate (*P = *0.002), and *N*-acetylasparagine (*P = *0.002). Intriguingly, inoculation with *L. iners* did not significantly alter metabolites related to proteinogenic amino acids or *N*-acetylated amino acids. These signatures from log-fold change and hierarchical clustering analyses were also observed in pairwise comparisons using one-way ANOVA (Fig. 7C).

**FIG 8:**
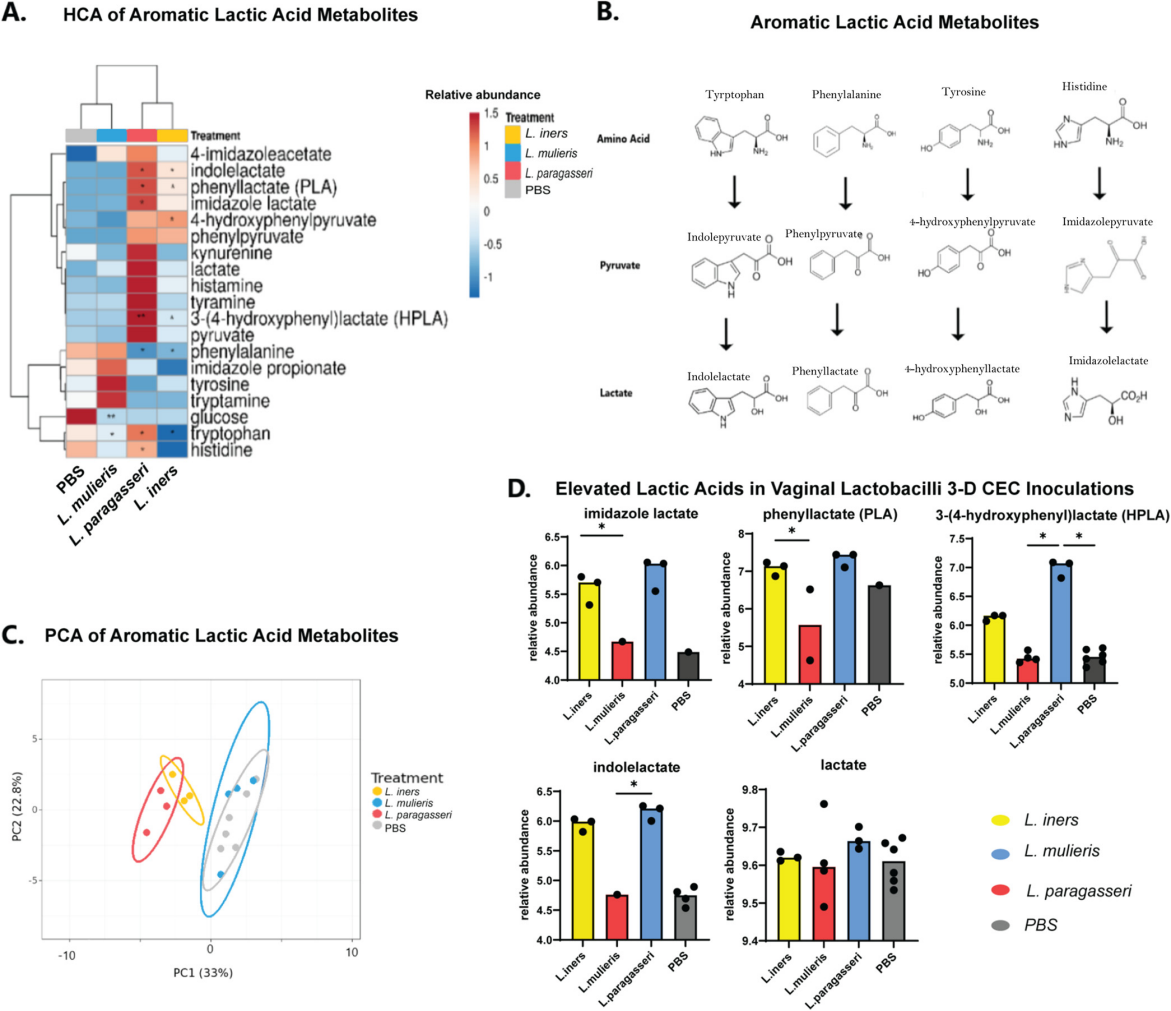
Vaginal *Lactobacillus* species elevated abundances of aromatic lactic acids. (A) Heatmap of metabolite relative abundance levels Hierarchical clustering was performed using Euclidean distance and Ward linkage. Asterisks indicate significantly altered metabolites in log-change analysis: *, *P < *0.05; **, *P < *0.01; ***, P < 0.001; ****, *P < *0.0001. (B) Diagram of four aromatic lactic acids measured in this study and their metabolic pathways from proteinogenic amino acids. (C) PCA of aromatic lactic acid metabolites showed distinct clustering between *L. iners* AB-107 and *L. paragasseri* JV-V03 and between *L. mulieris* JV-V16 and PBS mock-inoculated controls clustered together. (*D*) Boxplots of aromatic lactic acids and glucose-derived lactate compared among PBS mock-inoculated controls, *L. iners* AB-107, *L. mulieris* JV-V16, and *L. paragasseri* JV-V03. No significant differences in lactate abundance were observed between any lactobacilli evaluated and PBS controls based on Kruskal-Wallis test (*P* < 0.05). *, *P < *0.05; **, *P < *0.01; ***, *P < *0.001; ****, *P < *0.0001.

It is widely accepted that lactic acid is a key metabolite produced by lactobacilli that maintains homeostasis by lowering the pH of the vaginal environment (26). None of the lactobacilli significantly altered lactic acid metabolites, although *L. mulieris* significantly depleted glucose (*P = *0.01). Despite differences reported in the literature regarding the production of lactate from glucose among vaginal lactobacilli (71, 72), there were no significant differences between the vaginal lactobacilli and PBS controls in our study. However, aromatic lactic acids were significantly altered among the lactobacilli; therefore, to better understand aromatic lactic acid metabolism, we investigated upstream metabolites related to aromatic lactic acid production. Hierarchical clustering and PCA indicated that *L. paragasseri* and *L. iners* exhibit unique aromatic lactic acid profiles compared to *L. mulieris* and PBS controls (Fig. 8A and C). Unlike lactate, which is derived from glucose, these metabolites are derived from aromatic amino acids, such as tryptophan, phenylalanine, tyrosine, and histidine, that are converted to aromatic pyruvate derivatives followed by reduction to an aromatic lactic acid (Fig. 8B). In addition, *L. mulieris* modulated the relative abundance of tyrosine, tryptamine, and imidazole propionate but not imidazole lactate (Fig. 8A). *L. paragasseri* significantly elevated the relative abundance of HPLA (*P = *0.008), imidazole lactate (*P = *0.05), PLA (*P = *0.035), and ILA (*P = *0.048) relative to PBS controls. *L. iners* significantly elevated levels of ILA (*P = *0.046), PLA (*P = *0.046), and HPLA (*P = *0.031) relative to PBS controls (Fig. 8B). PCA illustrated the close relationship of *L. iners* and *L. paragasseri* regarding aromatic lactic acid metabolism, while *L. mulieris* clustered closely with PBS controls. PC2 accounted for 22.8% of the variance (*P = *0.002) and differentiated the species, while PC1 was indistinguishable (*P = *0.365). One-way ANOVA pairwise comparisons of aromatic lactic acids further supported the findings of both hierarchical clustering and log-fold change analysis, indicating elevation of lactic acids by vaginal lactobacilli (Fig. 8D). These results indicate a putative preferential production of amino acid-derived lactic acids by vaginal lactobacilli.

## DISCUSSION

Despite numerous studies on vaginal lactobacilli and their association with cervicovaginal health, there is still poor understanding of how specific lactobacilli species metabolically influence cervicovaginal homeostasis. The goal of this study was to identify distinct metabolic contributions in the cervicovaginal environment among key *Lactobacillus* species other than L. crispatus. This study also provided a context for other gut and vaginally relevant lactobacilli and limosilactobacilli that have close taxonomic and phylogenetic relationships with the strains investigated in this study. Further lactobacilli are abundant in the lower female reproductive tract (vagina and cervix); thus, we studied cervical host-microbe interactions using our well-characterized 3D CEC model (55, 73). CECs are the first line of defense for the host cervical mucosa (74). In addition, lactobacilli exert protective mechanisms in the cervicovaginal microenvironment and prevent the ascension of sexually transmitted pathogens to the upper reproductive tract (75, 76). Protection at this mucosal site may reduce the ascension of bacteria through the reproductive tract, uterine infections, and other gynecologic sequelae, such as preterm birth, pelvic inflammatory disease, endometritis, and cancer (7, 77, 78).

**FIG 9:**
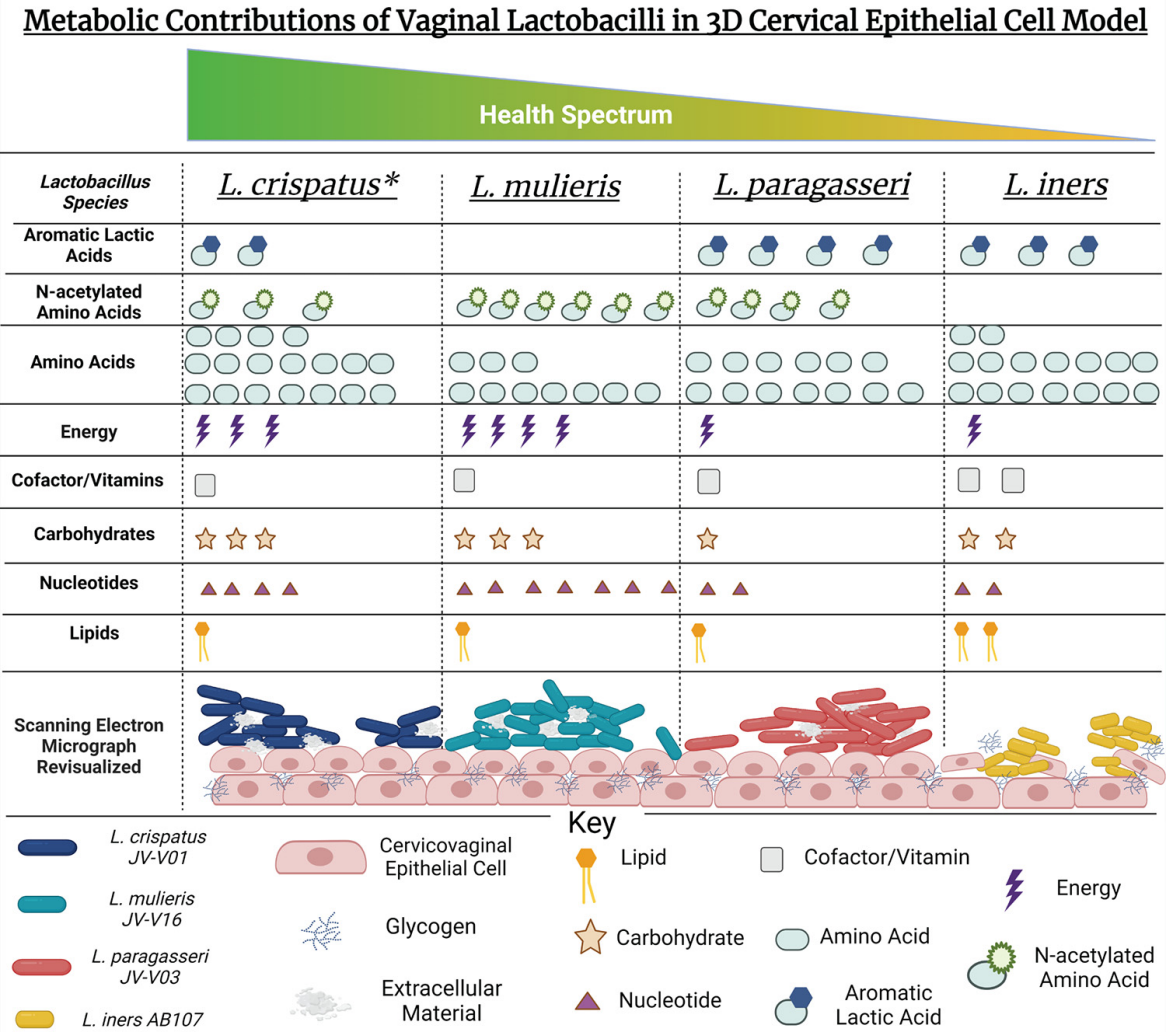
Synopsis of metabolomic results from 3D CEC models inoculated with L. crispatus JV-V01, *L. mulieris* JV-V16, *L. paragasseri* JV-V03, and *L. iners* AB-107. (A) L. crispatus JV-V01 was reported recently by our lab (47) and used as a key lactobacilli comparator. Amino acid metabolites were significantly altered compared to PBS controls by all strains of *Lactobacillus* sp. Aromatic lactic acids, derived from amino acids, were significantly elevated in cell culture supernatants of 3D CECs inoculated with L. crispatus, *L. iners*, and *L. paragasseri*. *N*-Acetylated amino acids were significantly accumulated in cell culture supernatants of 3D CECs inoculated with L. crispatus, *L. paragasseri*, and *L. mulieris.* These metabolites exhibited potential for antimicrobial capability as well as host immune stimulation, which may be how vaginal lactobacilli contribute to cervicovaginal homeostasis and inhibit ascension of BV-associated bacteria. Further SEM analysis revealed barrier disruption in 3D CECs inoculated with *L. iners* and an accumulation of lipids. This could signify why *L. iners* has been associated with both health and disease, as it may disrupt the barrier for host nutrients allowing other bacteria the ability to colonize. CECs inoculated with L. crispatus or *L. mulieris* also showed altered metabolites related to nucleotide and carbohydrate metabolism. Overall, vaginal lactobacilli share metabolic superpathways mostly in amino acid metabolism but are distinct in their individual metabolite signatures, even exhibiting novel metabolites that may contribute to maintenance of the cervicovaginal environment.

In this study, we evaluated three vaginal lactobacilli (*L. paragasseri*, *L. mulieris*, and *L. iners*). Each species has been implicated in modulating cervicovaginal health status (Table 1). Until recently, the strains utilized (*L. paragasseri* JV-V03 and *L. mulieris* JV-V16) were classified as some of the most common species in the vaginal environment: L. gasseri and L. jensenii, respectively (79–82). Additional studies are needed to assess the exact prevalence of *L. paragasseri*, L. gasseri, *L. mulieris*, and L. jensenii in cohorts using the latest taxonomic classifications. Here, we investigated the metabolic contributions of these well-characterized vaginal isolates in the cervicovaginal microenvironment using our robust 3D CEC model. This study complements our recently published metabolomics study that investigated the metabolomic influences of L. crispatus JV-V01 (vaginal isolate) and VPI 3199 (type strain) using our 3D CEC model (42) (Fig. 9).

Our genomic analyses of the selected strains validated previous findings on vaginal lactobacilli, such as taxonomic and phylogenetic lineage, genome size, and GC content differences between L. crispatus and *L. iners* (50, 57). The recent reclassification of the genus *Lactobacillus* (83, 84) to other genera, such as *Limosilactobacillus*, further complicates understanding the interactions between host and vaginal lactobacilli and how these microorganisms might benefit human health. However, it is clear that other rarer species identified from the vaginal environment (85–89), i.e., L. helveticus, L. acidophilus, *L. delbrueckii*, *L. delbrueckii* B, L. johnsonii, *L. fermentum*, *L. coleohominis*, *L. coleohominis* A, *L. vaginalis*, *L. reutri*, *L. reutri D*, and *L. reutri* E, require additional scientific attention. More studies are needed, with emphasis on these rare *Lactobacilliaceae* and their metabolic contributions and potential modulatory effects on the microbiome, especially as some family members are currently targets for use in probiotics therapies.

Additionally, genomic analyses support the hypothesis of putative host reliance of *L. iners* (29, 30, 50), indicated by *L. iners* encoding a lower number of genes related to metabolism than other lactobacilli. *L. mulieris* also exhibited low metabolic potential at the genomic level. However, *L. mulieris* modulated the broadest number of metabolites from multiple superpathways. These findings further emphasized the need to incorporate *in vitro* and *in silico* data together to illuminate the full metabolic picture of vaginal organisms in the cervicovaginal environment.

Using SEM, we confirmed that all vaginal lactobacilli assessed in this study effectively colonized 3D CEC models, thereby supporting the downstream analyses of host-microbe interactions. Adhesion of vaginal lactobacilli serves as a protective barrier against cervicovaginal colonization of other potentially harmful bacteria via competitive exclusion (90). Our findings of lactobacilli forming autoaggregates supported previous studies (91, 92) demonstrating that L. crispatus, L. gasseri, and *L. vaginalis* exhibit the greatest capability to form autoaggregates (93). In contrast, we observed signs of cell disruption in 3D CEC models inoculated with *L. iners.* This finding is supported by previous studies linking *L. iners* to increased cell permeability and cellular barrier disruptions (94), as well as secretion of a cytolysin (inerolysin) (95, 96).

*L. iners* and *L. paragasseri* demonstrated a high degree of similarity in metabolic profiles, supporting their close phylogenetic relationship. Amino acids were the most altered metabolites across all lactobacilli. Our *in vitro* work validated the same distinction of global metabolome profile clustering of vaginal lactobacilli in a clinical study (97). Furthermore, alterations in amino acid metabolism suggest the importance of amino acid availability for lactobacilli to produce antimicrobial peptides such as bacteriocins or other novel metabolites. Differentially, *L. mulieris* altered more metabolites related to energy and carbohydrate metabolism, similar to L. crispatus in our previous study (42), further supporting the phylogenetic relatedness between these two species. This is in accordance with clinical studies showing the metabolic characteristics of microbiomes dominated by L. crispatus or *L. mulieris* to be associated with optimal vaginal health (40). These findings suggest that although less common than L. crispatus, *L. mulieris* may be more important for optimal cervicovaginal health than previously appreciated. Carbohydrate metabolites were also observed to be altered by *L. iners*. Owing to the diversity of complex carbohydrates within the cervicovaginal environment, it might be advantageous for bacteria to utilize these carbohydrates as an energy source. Thus, bacteria capable of breaking down host glycans, such as *L. iners*, could not only outcompete other vaginal bacteria but also may be a way that lactobacilli contribute to carbohydrate cross-feeding in this environment (98, 99).

Previous studies have indicated that vaginal *Lactobacillus* species differ in amino acid biosynthesis (50). Additionally, genomic analyses of *L. iners* revealed this species is more reliant on host amino acids than is L. crispatus (50, 100). Our amino acid metabolomic data for *L. iners* compared to mock-inoculated controls indicated enrichment of amino acids. However, further experiments are needed to determine host or bacterial origin of these metabolites derived. Although all lactobacilli isolated from our study altered metabolites related to amino acid metabolism, their amino acid metabolic profiles differed among each species. Lactic acid bacteria are known to produce branched-chain amino acids (41). Clinical studies have found that high concentrations of valine, leucine, and isoleucine are correlated with a high prevalence of lactobacilli in healthy pregnant women (101, 102). Interestingly, our study indicated that, relative to mock-inoculated controls, all lactobacilli produced levated levels of branched-chain amino acids or related metabolites, such as 2-hydroxy-3-methylvalerate and alpha-hydroxyisocaproate, which are putative antimicrobial properties (103). Others have observed metabolites such as glycerophospholipids being positively correlated with *Lactobacillus* abundance (104). We also observed an accumulation of glycerophospholipids in response to 3D CEC inoculation with *L. iners* and *L. mulieris*. It is thought that in an evolutionary context, the abundance of glycerophospholipids can be linked to the close relationship between a eukaryotic host and its symbiotic bacterial counterpart (105).

In healthy reproductive-age women, vaginal metabolomes exhibit elevated levels of lactic acid and elevated concentrations of amino acids, including isoleucine, leucine, tryptophan, phenylalanine, aspartate, glutamine, and pi-methylhistidine, which correlate with lactobacilli abundance (40, 41, 106–108). Tryptophan, phenylalanine, histidine, and tyrosine are all known to be precursors to aromatic lactic acids. Aromatic lactic acids are lactate derivatives synthesized from aromatic amino acids instead of glucose. The aromatic structure in bacterially derived metabolites has recently been a novel factor in many beneficial host modulations. Kynurenine, a precursor to HPLA, was a distinguishable biomarker for *Lactobacillus*-dominated vaginal profiles compared to patients with BV (108). Our study further supported these findings by demonstrating that *L. iners*, *L. paragasseri*, and *L. mulieris* metabolic profiles were highly enriched in amino acid metabolites and aromatic lactic acids (Fig. 6 to 8). Aromatic lactic acids were originally identified as antifungal inhibitory compounds in the fermentation and preservation of food products (109). Additionally, these lactic acids play a role in gut homeostasis, from lactic acid bacteria such as bifidobacteria and lactobacilli (110). Fecal phenolic-derived metabolites have also been linked to health and are positively correlated with lactobacilli abundance (111). Interestingly, elevated concentrations of the putative antimicrobial compound PLA were also observed among BVAB and L. crispatus in a recent study (42). Imidazole lactate, however, was unique to L. crispatus in a study by Laniewski and Herbst-Kralovetz, which was also observed in *L. mulieris* in this study. Recent work has established the role of PLA as an inhibitory compound against urinary pathogens in supernatants from L. crispatus isolated from the bladder (112). Further research is required to define the contributions of these aromatic lactic acids in terms of whether there are differences in l- and d-isoforms of these metabolites and their abilities to inhibit genital pathogens, as well as modulate the host cell and immune response.

*N*-Acetylated amino acids are of particular interest, as acetylation may protect against enzymatic degradation in low-pH environments (113). Due to the acidification of the environment by lactobacilli, this could explain the elevation of *N*-acetylated amino acids in the presence of *Lactobacillus* species, as amino acids may be necessary for other downstream protein pathways. Although our understanding of *N*-acetylation processes by commensal bacteria is limited, previous research indicated that these metabolites exhibit putative antimicrobial capability and serve as host-interactive molecules, which suggests a similar function in the cervicovaginal microenvironment (114–116). These metabolites, including *N*-acetylarginine, *N*-acetylserine, and *N*-acetylthreonine, were also identified in our previous *in vitro* study to be elevated following L. crispatus colonization (42). These additional compounds could play a role in cervicovaginal health; however, their mechanistic actions remain to be elucidated.

The limitations of this study are that vaginal *Lactobacillus* taxonomic reclassification was performed at the time of experimentation. Due to this, we utilized formerly classified strains to L. jensenii (*L. mulieris*) and L. gasseri (*L. paragasseri*); however, further studies need to be conducted on strains that are still classified as L. gasseri and L. jensenii (79, 82). This study provided a foundation for additional inquiry into other rare lactobacilli or limosilactobacilli that have been identified in vaginal microbiome samples and whether taxonomic lineage truly does match beneficial metabolic properties. Despite these limitations, our experimentation utilized well-characterized *Lactobacillus* spp. strains isolated from the female genital tract that have been used in many *in vitro* studies, therefore providing better cross-comparison of results (44, 46, 57). Further, this study yielded reproducible results in multiple biological replicates. Similarly, three other recent studies using the same experimental model have been published on other key vaginal bacteria (42, 117, 118). Those studies provided further context to physiological mechanisms that are shared and unique among vaginal microbes and their relationship to health and disease. Future studies should be completed on multiple strains to better gauge possible variability or lack thereof in metabolomic profiles. In addition, studies utilizing multiple *Lactobacillus* species and bacterial mixed cultures (e.g., *L. iners* with BVAB) will advance our understanding of host-microbiome interactions.

In this study, we identified key metabolic differences between three common cervicovaginal lactobacilli. Lipid metabolism and cofactor and vitamin metabolites were more associated with *L. iners*. In contrast, energy and carbohydrate metabolism were more associated with *L. mulieris*, and overall amino acid metabolites were a signature from all tested vaginal *Lactobacillus* species. This study highlighted key metabolites, such as *N*-acetylated amino acids and aromatic lactic acids, that could play critically important roles in maintaining health and homeostasis, as well as, defense of the microenvironment via competitive exclusion and protection from colonization and ascension of pathogenic bacteria to the upper female reproductive tract. Coupling our 3D CEC model with metabolomics approaches allowed us to identify key metabolites that distinguish vaginal lactobacilli, as well as lead to the identification of putative antimicrobial metabolites. Future studies can utilize the metabolites identified in this study to further determine their beneficial features, interactions with other vaginal bacteria, including BVAB, and their direct effects on host cells. Furthermore, these metabolites could serve as novel postbiotic therapies.

## MATERIALS AND METHODS

### Culture of the 3D human CEC model.

Human cervical epithelial cells (A2EN) (119) were grown with keratinocyte serum-free medium (Thermo Fisher Scientific, Waltham, MA, USA) supplemented with epidermal growth factor (5 ng/mL; Thermo Fisher Scientific, Waltham, MA, USA), sodium chloride (22 mg/mL; Sigma-Aldrich, St. Louis, MO, USA), bovine pituitary extract (50 μg/mL; Thermo Fisher Scientific, Waltham, MA, USA), and Primocin (100 μg/mL, InvivoGen, San Diego, CA, USA) in a humidified atmosphere of 5% CO_2_ at 37°C as previously reported (73). Before seeding, cells were enumerated using the Countess automated cell counter (Invitrogen) and trypan blue exclusion. To generate the 3D CEC model, human cervical epithelial A2EN cells were grown on Cytodex-3 collagen-coated dextran microcarrier beads (Sigma-Aldrich) in a rotating wall vessel bioreactor (Synthecon), as previously described (53–55). Cells were incubated for a 28-day differentiation period with continuous rotation at 20 rpm and provided with fresh medium daily after an initial 3-day period without fresh medium to allow cells to adhere and establish aggregates. After the 28-day differentiation period, 3D CEC aggregates were seeded into 24-well culture-treated plates at the density of 1 × 10^5^ to 5 × 10^5^ cells/mL and used for downstream analyses.

### Bacterial strains and growth conditions.

*L. paragasseri* strain JV-V03 and *L. mulieris* strain JV-V16 were cultured on De Man, Rogosa and Sharpe agar (Thermo Fisher Scientific, Waltham, MA, USA). *L. iners* AB-107 was cultured on tryptic soy agar (Thermo Fisher Scientific, Waltham, MA, USA) supplemented with 5% (vol/vol) defibrinated sheep blood (Quad Five, Ryegate, MT). Bacteria were grown at 37°C under anaerobic conditions and generated using AnaeroPack-Anaero anaerobic gas generator and jars (Thermo Scientific, Waltham, MA, USA). *L. paragasseri* strain JV-V03 and *L. mulieris* strain JV-V16 were obtained from the Biodefense and Emerging Infections Research Resources Repository (https://www.beiresources.org) (Table 1). *L. iners* strain AB-107, also known as ATCC 55185, was obtained from ATCC Bioresources Center (https://www.atcc.org/) (Table 1). *L. paragasseri* strain JV-V03 and *L. mulieris* strain JV-V16 were isolated from the healthy female urogenital tract, and *L. iners* strain AB-107 was isolated from a patient with BV. These strains are well-characterized representatives of vaginal lactobacilli (43, 46, 120). Partial genomic sequences were available at NCBI (https://www.ncbi.nlm.nih.gov/genome/) for all three strains. Updated taxonomy was validated by Genome Taxonomy Database (https://gtdb.ecogenomic.org/), which is the most up-to-date taxonomic lineage for *L. paragasseri* JV-V03, formerly L. gasseri (79, 80, 121), and *L. mulieris* JV-V16, formerly L. jensenii (81, 82).

### Lactobacilli coculture with cervical epithelial cells.

Before inoculation, bacteria were grown for 16 to 18 h on the appropriate agar medium, as described above. Bacteria were harvested, resuspended in sterile Dulbecco’s phosphate-buffered saline (PBS), and adjusted to an optical density at 600 nm (OD_600_) of 0.5. Bacterial viability was confirmed using a standard plating assay. To enumerate CFU, the adjusted bacterial cell suspensions were serially diluted in PBS, plated on the respective agar for growth, and incubated for 96 h at 37°C under anaerobic conditions. The 3D CECs were inoculated with bacterial suspensions (20 μL) of an OD_600_ of 0.5, which corresponded to approximately (1 to 2) × 10^5^ CFU/mL for 24 h at 37°C under anaerobic conditions, as described previously (108).

### Taxonomic classification and phylogenetic analysis of species from the genera *Lactobacillus* and *Limosilactobacillus*.

To better understand the relationships of *Lactobacillus* species and the strains included in this study, incorporate genomic trees were constructed. It is important to note that recent reclassification of the genus *Lactobacillus* led to discrepancies between well-known databases, such as NCBI. Due to this reclassification, the taxonomic relationships of *Lactobacillus* species were analyzed utilizing the tool PhyloT version 2022.3 (122), which is integrated to work with two public databases: NCBI and GTDB release 207.0 (123). The first generated tree included strains from the NCBI Taxonomy browser (124), which identifies taxonomic lineages of NCBI genomes. This tree incorporated lactobacilli species that are most prevalent in the vaginal microbiome: *L. iners* (16 strains), L. crispatus (25), L. gasseri (20), *L. paragasseri* (2), and L. jensenii and *L.mulieris* (20). NCBI databases do not yet fully recognize the L. jensenii-*L. mulieris* distinction despite literature published on the separation of these species (81, 82).

Since *Lactobacillus* reclassification in NCBI is not up to date with these reclassifications, GTDB was additionally utilized. The GTDB-constructed tree included species from *Lactobacillus* and *Limosilactobacillus* to provide a broader picture of phylogenetic relationships of these genera, including 926 strains from *Lactobacillus* species (32 *L. iners strains*, 161 *L. crispatus strains*, 41 *L. gasseri strains*, 40 *L. paragasseri strains*, 31 *L. jensenii strains*, and 18 *L. mulieris strains*) and 452 strains from limosilactobacilli. *Limosilactobacillus* was included in the analysis, since the recent restructuring of the genus *Lactobacillus* (83, 84) included several species of *Limosilactobacillus* identified in vaginal microbiome samples, such as *L. vaginalis* (8 strains), L. reuteri (225 strains), *L. fermentum* (102 strains), and *L. coleohominis* (9 strains), which have also been identified in vaginal microbiome samples. Taxonomic and phylogenetic trees were visualized and edited in iTOL version 6.6 (125).

### Genomic annotation of *Lactobacillus* strains.

To better understand the contributions of these vaginal *Lactobacillus* isolates, L. crispatus was included in genomic analyses. This species and the ones discussed are the lactobacilli most observed observed in the vaginal environment. L. crispatus strain JV-V01 is a highly characterized strain, and recent metabolomic data of this strain in the 3D cell model are available in Laniewski et al. (42). DNA sequences of *L. iners* AB-1 (ATCC 55195), *L. mulieris* JV-V16, *L. paragasseri* JV-V03, and L. crispatus JV-V01 were obtained from NCBI’s GenBank database (https://www.ncbi.nlm.nih.gov/genbank/). FASTA format sequences were uploaded to PATRIC (Pathosystems Resource Integration Center integration of RAST, the Rapid Annotation Server) (68, 126, 127) for genome annotation.

### Comparative genomic analysis of *Lactobacillus* strains.

To identify the similarity of predicted genes, FASTA-formatted DNA sequences were uploaded to OrthoVenn2 (https://orthovenn2.bioinfotoolkits.net/home) (65). OrthoVenn2 identifies orthologous gene clusters using Markov clustering models and BLASTp alignments, as described previously (128). To identify the differences in overall predictive genomic functionality, a number of genes within subsystems defined by PATRIC (68) were observed and then statistically analyzed. For statistical comparison of these subsystems, 26 bacterial strains with whole-genome sequences from the four *Lactobacillus* species, L. crispatus, L. iners, L. paragasseri, and L. mulieris-L. jensenii were selected from the GenBank database (https://www.ncbi.nlm.nih.gov/genbank/) as of 1 October 2021. The ggplot2 package visualized grouped bar plots of these data in R version 4.1.2 (https://www.r-project.org/). To analyze the unique predicted protein profiles of the strains of interest, the PATRIC protein sorter was utilized (68). Three discrete categories were made: hypothetical proteins, proteins with known metabolic function, and proteins with nonmetabolic functions. Donut plots of these profiles were visualized with the Canva program.

### Cytokine, chemokine, and growth factors multiplex analysis.

Cell culture supernatants were collected from at least three independent replicates of 3D human CECs inoculated with the bacteria described above and used to quantify concentrations of 15 protein targets. PBS-treated cells served as negative controls, and Atopobium parvulum, taxonomically reclassified as *Lancefieldella parvula*, was used as a positive control. Positive control data were obtained simultaneously, and culture conditions are those described in Maarsingh et al. (129). Cytometric bead arrays were performed using customized MILLIPLEX multianalyte profiling human cytokine and chemokine panel 1, in accordance with manufacturer guidelines. The tested targets included fractalkine, IL-1α, IL-1β, IL-6, IL-8, IP-10, MCP-1, MCP-3, MIP-1β, PDGF-AA, RANTES, TGF-α, TNF-α, and VEGF. Data were collected and analyzed using a BioPlex 200 platform and BioPlex Manager (5.0) software (Bio-Rad). A 5-parameter logistic regression curve fit was used to determine concentrations. All samples were assayed in duplicate.

### Untargeted global metabolomics.

Cell culture supernatants were collected from four independent 3D CEC aggregate batches inoculated with *L. paragasseri* JV-V03, *L. iners* AB-107, or *L. mulieris* JV-V16, along with eight PBS-treated controls, and were sent to Metabolon Inc. (Durham, NC) for untargeted global metabolomics analysis. Metabolites were resolved on a Waters ACQUITY ultraperformance liquid chromatography system (UPLC) and a Thermo Scientific Q-Exactive high-resolution accurate mass spectrometer interfaced with a heated electrospray ionization (HESIII) source and Orbitrap mass analyzer operated at 35,000 mass resolution. The sample extract was dried and then reconstituted in solvents compatible with the four methods. Each reconstitution solvent contained a series of standards at fixed concentrations to ensure injection and chromatographic consistency. One aliquot was analyzed under acidic positive ion conditions, chromatographically optimized for more hydrophilic compounds. In this method, the extract was gradient eluted from a C_18_ column (Waters UPLC BEH C_18_, 2.1 by 100 mm, 1.7 μm) using water and methanol containing 0.05% perfluoropentanoic acid (PFPA) and 0.1% formic acid (FA). Another aliquot was also analyzed using acidic positive ion conditions. In this method, the extract was gradient eluted from a C_18_ column using methanol, acetonitrile, water, 0.05% PFPA, and 0.01% FA and was operated at an overall higher organic content. Another aliquot was analyzed using basic negative ion optimized conditions using a separate resolute C_18_ column. The basic extracts were gradient eluted from the column using methanol and water, but with 6.5 mM ammonium bicarbonate at pH 8. The fourth aliquot was analyzed via negative ionization following elution from a HILIC column (Waters UPLC BEH amide, 2.1 by 150 mm, 1.7 μm) using a gradient of water and acetonitrile with 10 mM ammonium formate, pH 10.8. The mass spectrometry (MS) analysis alternated between MS and data-dependent tandem mass spectrometry scans using dynamic exclusion. The scan range varied slightly between methods but covered 70 to 1,000 *m/z*. The bioinformatics system consisted of four major components, the Laboratory Information Management System (LIMS), the data extraction and peak identification software, data processing tools for quality control (QC) and compound identification, and a collection of information interpretation and visualization tools for use by data analysts. Peaks were quantified using area under the curve. Although proprietary techniques established by Metabolon Inc. can identify a wide array of metabolites, these techniques are currently unable to identify isomeric forms of metabolites. Three batches, two from *L. paragasseri* JV-V03 and one from *L. iners* AB-107, and respective PBS control samples were removed due to global outlier values determined by MetaboAnalyst 5.0 (https://www.metaboanalyst.ca/).

### Statistical analysis.

Genomic statistical analyses were performed using the Kruskal Wallis test in R version 4.1.2 (https://www.r-project.org/). All inoculations and assays were performed as at least three independent replicates. One-way ANOVA with Dunnett’s adjustment for multiple comparisons was used to statistically analyze the immunoproteomics data in Prism v8 software (GraphPad). Differences in metabolite pathway composition were determined by chi-squared analysis. Hierarchical clustering analysis and heatmap visualization were performed using ClustVis (https://biit.cs.ut.ee/clustvis/) (130) with Euclidean distance and Ward linkage. Metabolite intensity values were median scaled and log transformed prior to performing two-tailed paired Student’s *t* tests (lactobacilli inoculated versus PBS control). One-way ANOVA was performed using MetaboAnalyst 5.0 (https://www.metaboanalyst.ca/) (131), which is integrated with R statistical software and has been validated by Prism v8 software (GraphPad). Principal-component analysis, Pearson’s correlation analysis, and metabolite pathway enrichment analysis were performed in MetaboAnalyst 5.0 (https://www.metaboanalyst.ca/) (131). *P* values of <0.05 were considered significant in all analyses. Metabolomics results were corrected for multiple testing using the Bonferroni false-discovery rate, and *q* values were reported. All error bars represent standard deviations.

### Data availability.

Data supporting the findings of this study are available within the paper and supplemental material; any additional data can be requested from the corresponding author.
