# Supplementary figures and images for: Commensal Lactobacilli Metabolically Contribute to Cervical Epithelial Homeostasis in a Species-Specific Manner
Source: mSphere. 2023 Jan 11;8(1):e00452-22. doi: 10.1128/msphere.00452-22 (PMC9942568; doi:10.1128/msphere.00452-22)

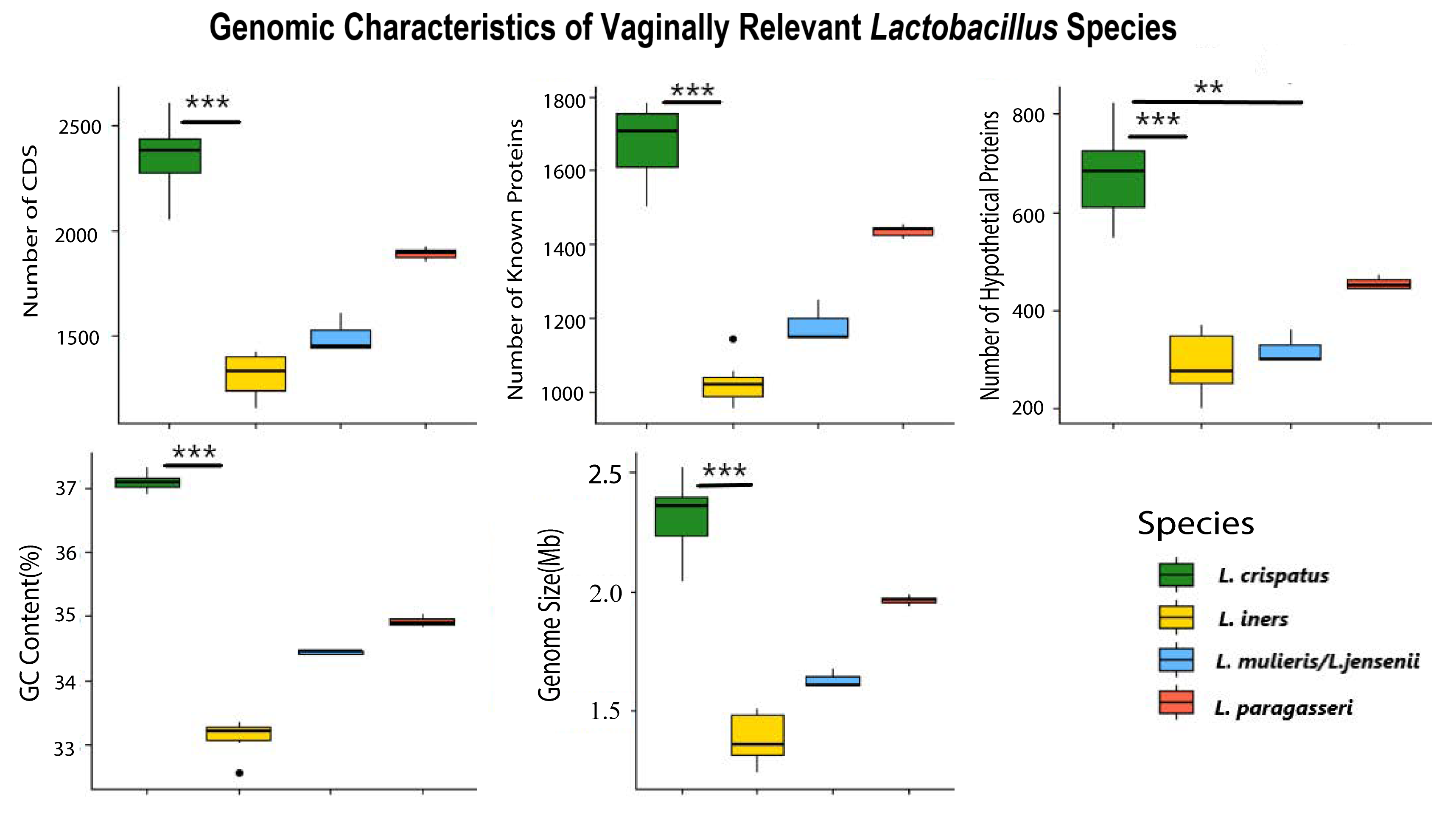

Supplement: FIG S1 [file msphere.00452-22-s0006.tif]

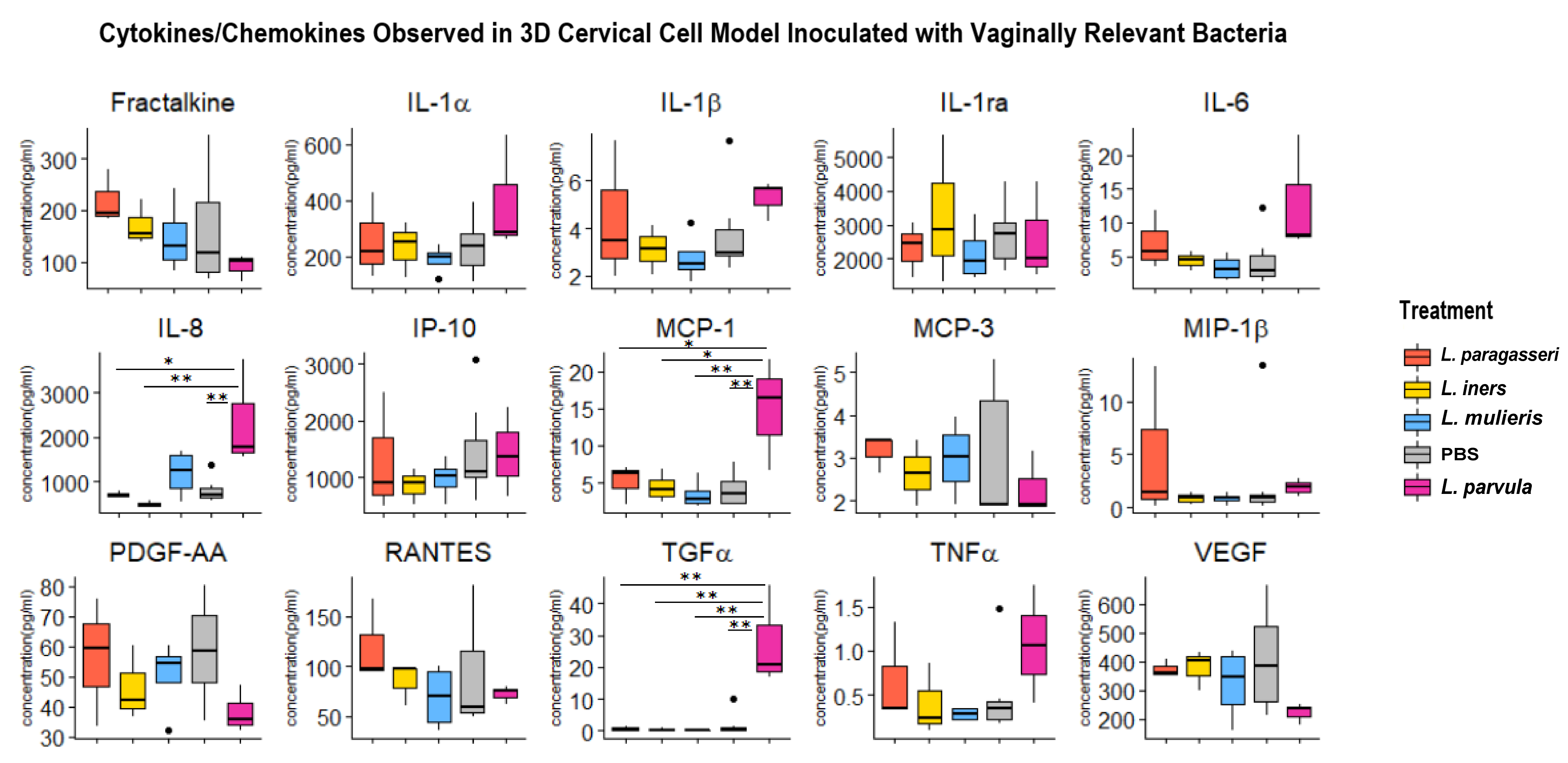

Supplement: FIG S2 [file msphere.00452-22-s0007.tif]

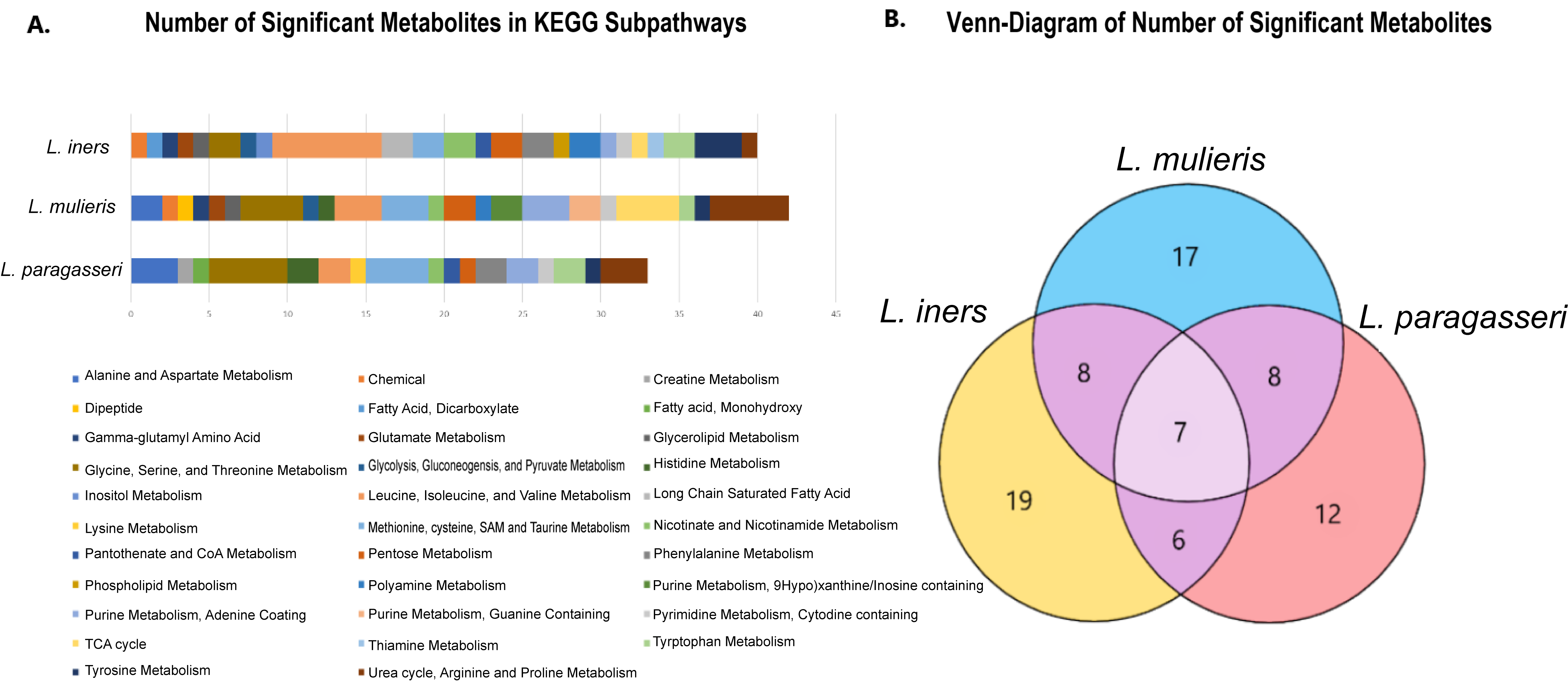

Supplement: FIG S3 [file msphere.00452-22-s0008.tif]

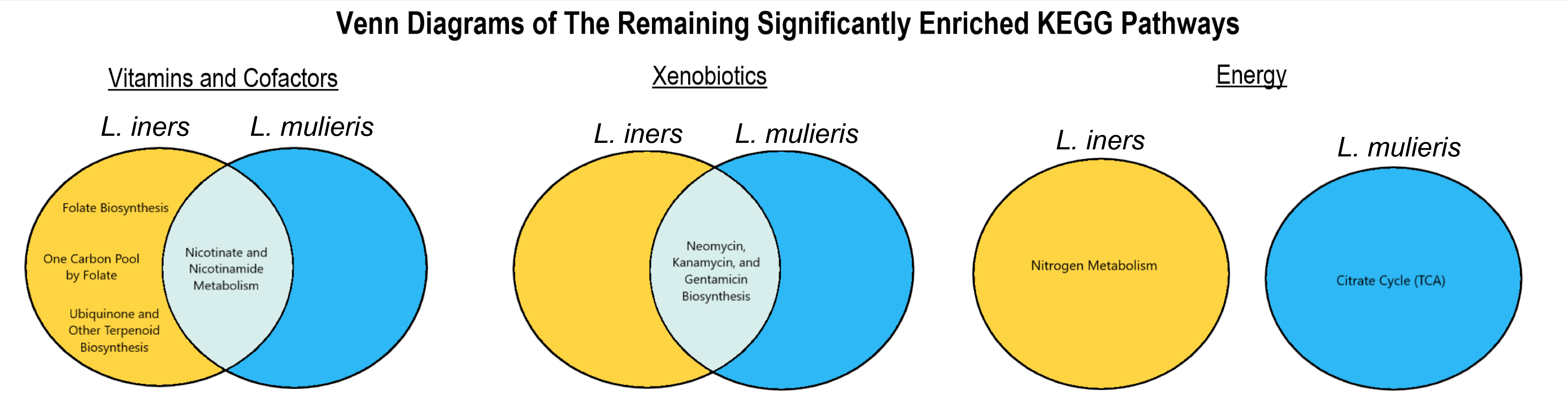

Supplement: FIG S4 [file msphere.00452-22-s0009.tif]
